# siRNA-Silencing of *swnR* gene greatly reduced biosynthesis of swainsonine in *Alternaria oxytropis* without affecting the growth characteristics of leguminous host

**DOI:** 10.3389/fmicb.2025.1641192

**Published:** 2025-08-25

**Authors:** Yu Zhang, Liwen Yang, Yange Li, Shiyu Tang, Yiqingqing Zhang, Pinzhi Sun, Hao Lu

**Affiliations:** College of Veterinary Medicine, Northwest A&F University, Xianyang, Shaanxi, China

**Keywords:** locoweed, *Alternaria oxytropis*, swainsonine, siRNA, transcriptome, metabolome, fungi and plants interact

## Abstract

Locoism refers to a neurological disorder in livestock caused by chronic ingestion of locoweeds, which contain toxic alkaloid swainsonine produced by the fungus *Alternaria oxytropis*. Therefore, reducing swainsonine levels not only prevents locoism but may also transform these toxic plants into animal feed. In this study, we identified a pivotal role for the *swnR* gene in swainsonine biosynthesis. Using siRNA-mediated gene silencing, we demonstrated that knockdown of *swnR* markedly reduced swainsonine accumulation in fungal mycelia. Transcriptomic and metabolomic analyses revealed that *swnR* silencing triggered broad metabolic reprogramming, notably impacting aromatic amino acid metabolism, carbon metabolism, and antioxidative pathways, and underscoring its central role in fungal growth and secondary metabolism. Furthermore, we screened and inoculated a hypovirulent strain suitable for co-cultivation with peeled seed embryos of *Oxytropis glabra*, and the symbiont showed significantly reduced level of swainsonine without negatively impacting plant growth. These findings provide a promising strategy for mitigating locoism by engineering endophytic fungi with attenuated toxicity.

## 1 Introduction

Locoweed refers to specific toxic plants within the genera *Oxytropis* and *Astragalus* of the legume family. The locoweed species are widely distributed across several regions, including North America (Mexico, Canada, and the United States) (Ralphs et al., 2002; Noor et al., 2020), parts of Asia (China, Pakistan, India, and Mongolia) (de Carvalho Nunes et al., 2019; Wu et al., 2016), South America (Argentina, Brazil and Chile) (Dantas et al., 2007; Mendonça et al., 2012; Micheloud et al., 2017), and certain areas in Western Europe. Locoweed is characterized by its well-developed root systems, dense foliage, and strong resistance to adverse environmental conditions, including drought, cold, pests, and diseases (Ralphs et al., 2008; Zhuang et al., 2015; Guan et al., 2019; Cholich et al., 2021). Due to their hardiness, these plants can outcompete with high-quality forage for vital resources such as soil nutrients, water, and sunlight resulting in their excessive proliferation over other vegetation that disrupts the ecological balance of grasslands and reduces the overall utility of these areas (Vignale et al., 2013; Pérez et al., 2013).

Livestock that graze on or inadvertently ingest locoweed in sufficient quantities can experience chronic poisoning, which manifests primarily as neurological dysfunction. In severe cases, this poisoning can lead to death, resulting in significant economic losses for pastoral industries (Wang et al., 2024).

Extensive evidence indicates that the primary cause of livestock poisoning from locoweed is the toxic alkaloid swainsonine generated by the endophytic fungus *A. oxytropis* (Lu et al., 2015). When this endophyte is removed, locoweed produces little to no swainsonine (Moremen, 2002; Braun et al., 2003; Bedin et al., 2010; Lu et al., 2014). Swainsonine exerts its toxic effects by inhibiting lysosomal α-mannosidase activity, leading to disruptions in oligosaccharide metabolism and, ultimately, causing widespread vacuolar degeneration in cells (Oldrup et al., 2010; Grum et al., 2012; Cook et al., 2013).

**Figure 1:**
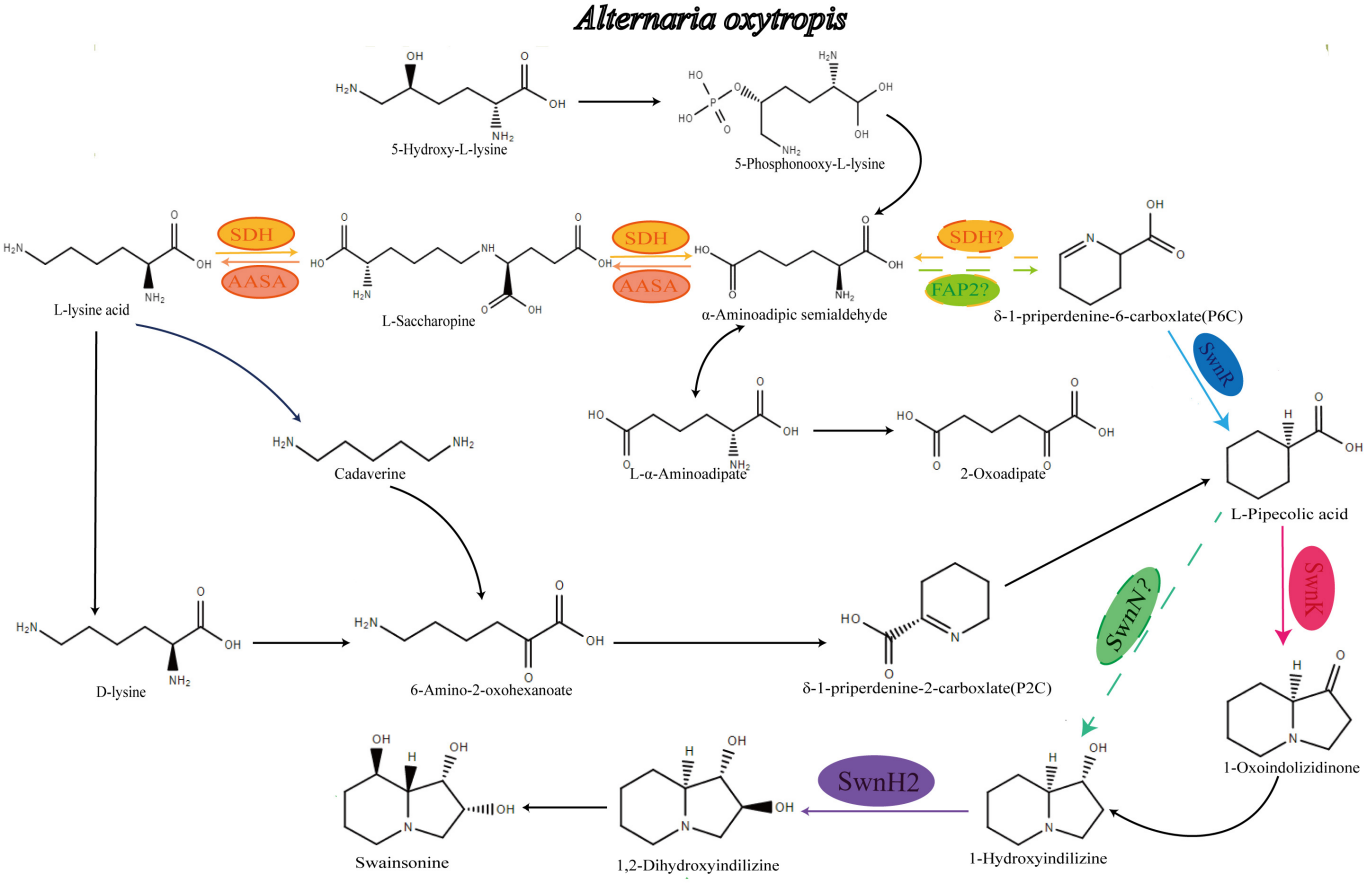
Speculative synthetic pathway of *A. oxytropis* to produce swainsonine. AASA, α-aminoadipate hemialdehyde synthase; *sdh*, Saccharopine dehydrogenase; *fap2*, Saccaropine oxida.

In addition to *A. oxytropis*, certain fungi from the genus *Metarhizium* (Luo et al., 2020; Huang et al., 2023), the clover pathogen *Slafractonia leguminicola* (Alhawatema et al., 2025; Das et al., 2023), symbionts of *Ipomoea carnea* (Neyaz et al., 2022; Cholich et al., 2023), and fungi from the order Chaetothyriales (Cholich et al., 2023) are also known to produce swainsonine. To date, extensive research has been conducted on the swainsonine biosynthetic pathways in *S. leguminicola* and *Metarhizium species* (Sim and Perry, 1997; Singh and Kaur, 2012; Cook et al., 2017; Luo et al., 2020), but the biosynthetic pathway in *A. oxytropis* remains largely unexplored. Current evidence suggests that lysine and L-pipecolic acid serve as common precursors and intermediates in the swainsonine biosynthesis of these fungi. Studies have identified a shared gene cluster, referred to as “*SWN*,” in swainsonine-producing fungi such as *A. oxytropis, M. anisopliae*, and *S. leguminicola*. This cluster includes the genes *swnH1, swnH2, swnK, swnN, swnR, swnA*, and *swnT*, which are believed to encode the enzymes required for the conversion of L-pipecolic acid into swainsonine (the predicted path diagram is shown in the Figure 1) (Cook et al., 2017). Further research has revealed that while the *swnT* gene mediates toxin transport in *S. leguminicola*, it does not appear to facilitate swainsonine transport and secretion in *M. robertsii*. This suggests that the catalytic enzyme genes within the *SWN* gene cluster may have strain-specific functional roles (Luo et al., 2020; Das et al., 2023).

This study identifies *swnR* as essential for swainsonine biosynthesis in *A. oxytropis*. A *swnR*-silenced mutant co-cultured with *O. glabra* formed a hypovirulent symbiont that eliminates livestock toxicity without compromising host viability, enabling safer locoweed utilization.

## 2 Materials and methods

### 2.1 Fungal strains and culture conditions

In this study, wild-type strains (WT) were isolated from oxytropis glabra in Gansu Province and analyzed by PCR detection (primers were ITS1: 5′TCCGTAGGTGAACCTGCGG 3′/ITS4: 5′TCCTCCGCTTATTGATATGC 3′, respectively), and through NCBI Blast, it was found that the sequence of this strain was 100% similar to Alternaria sp. ZC-2014 (a type of *Alternaria oxytropis*) (Woudenberg et al., 2013). This fungus needs to be routinely cultivated on PDA (potato dextrose agar) media at a temperature of 20°C.

### 2.2 Plant materials and culture conditions

Seeds previously collected from *Oxytropis glabra* plants (Jiuquan, Gansu, coordinates) were used for all experiments described below. The method of seed coat removal was employed to qualitatively reduce the amount of *A. oxytropis* inoculum. Additionally, seed embryos without seed coats were inoculated into fungal cultures. Plants derived from these seeds or embryos were cultivated in an artificial climate chamber under a 16-h light cycle, with day/night temperatures maintained at 20°C/18°C.

### 2.3 Detection of swainsonine content in fungal mycelia

*Alternaria oxytropis* was inoculated onto PDA plates and cultured at 20°C. Mycelia was collected daily from the 6th to the 10th day, three repetitions were set for each group. After drying, the mycelia mass was measured, and it was promptly ground into a fine powder using a grinder, and then the mycelia is extracted with methanol (the method was consistent with Song et al., 2019). The mycelia was analyzed using Triple Quad 5500+QTRAP Ready and using the methods of (Noor et al., 2020). SW concentration was tested three times for each strain.

### 2.4 Detection of swainsonine content in fungal culture mycelia

*Alternaria oxytropis* was inoculated onto PDA plates and cultured at 20°C. The culture media was collected daily from the 6th to the 10th day. The solid media was chopped into small pieces and soaked in 400 mL of methanol overnight. After soaking, ultrasonic extraction was carried out for 60 minutes, repeated three times. Combined with solvent evaporation and concentration, the crude extract was obtained and dissolved with methanol. Three parallel replicates were set up for each strain. The specific detection method is the same as that for the “Section 2.3” in the previous section.

### 2.5 Fungal RT qPCR gene expression levels and correlation analysis

#### 2.5.1 RNA extraction and cDNA synthesis

*Alternaria oxytropis* was cultured on PDA at 20°C. Fresh mycelia (days 6–10 post-inoculation) were homogenized in 1 mL TRIZOL reagent (Solarbio) using a tissue grinder (1 min), incubated on ice (15 min), and mixed with 200 μL chloroform (VWR Chemicals). After phase separation by centrifugation (14,400 g, 15 min, 4°C), 500 μL of the aqueous phase was combined with 500 μL isopropanol (VWR Chemicals), incubated on ice (15 min), and centrifuged (12,000 g, 15 min, 4°C). The RNA pellet was washed twice with 75% ethanol (anhydrous ethanol/DEPC-treated water), air-dried, and resuspended in 30 μL DEPC-treated water. RNA integrity was verified by 1% agarose gel electrophoresis, concentration and purity (A_260_/A_280_) were measured spectrophotometrically.

High-quality RNA (4,000 ng) was reverse-transcribed into cDNA using the YEASEN kit (11141ES60), including a no-reverse-transcriptase control. The resulting cDNA was stored at −20°C until further use.

#### 2.5.2 qPCR analysis

Gene-specific primers (Supplementary Table S2) were designed using Primer 7.0 and validated for specificity (NCBI Primer-BLAST), amplification efficiency (>80%), and absence of dimers (melt curve analysis). Reactions (20 μL) contained 200 nM primers, cDNA (80 ng RNA input), and Deyi qPCR master mix. All samples/controls (no-template, no-RT) were run in triplicate on a Roche LightCycler 96 under standard conditions: 40 cycles of 95°C (10 s), 60°C (1 min; combined annealing/extension), followed by melt curve analysis.

### 2.6 Preparation of the pSilent-*swnR* silencing vector

The pSilent-*swnR* vector was constructed in two steps using the pSilent-1 plasmid. Based on the sequence of the *swnR* gene from *A. oxytropis* (KY365742.1), primers were designed to amplify two fragments, a 332 bp forward and reverse fragment of the *swnR* gene, each including homologous arms (primer sequences are listed in Supplementary Table S3). The pSilent-1 plasmid was first digested with *Kpn* I/*Bgl* II and then subjected to double digestion with *Xho* I/*Hind* III. The *swnR* gene fragments 1 and 2 were successfully amplified. These two *swnR* gene fragments were then ligated into the enzyme-digested pSilent-1 plasmid fragment. The ligation product was transformed into competent cells, and positive transformants were selected. Sequencing was performed on the transformants, and those with sequences completely matching the target *swnR* fragment were identified as containing the pSilent-*swnR* silencing vector. These positive transformants were used for further experimentation.

### 2.7 Preparation of protoplasts

*A. oxytropis* was inoculated onto PDA plates and cultured at 20°C for 6–10 days. The mycelia was scraped off, washed with an osmotic stabilizer (1.2 M KCl), and centrifuged. The centrifuged *A. oxytropis mycelia* was weighed, and different enzymatic digestion solutions were prepared with varying enzyme combinations at a solid-to-liquid ratio of 1:10. The enzyme combinations tested included: 1% cellulase + 3% lysing enzyme, 1% snailase + 3% lysing enzyme, 1% cellulase + 1% snailase, and 1% snailase + 1% cellulase + 3% lysing enzyme. The 50 mL centrifuge tubes containing the enzymatic digestion solutions were wrapped in aluminum foil to protect them from light and placed on a shaker at different temperatures (20°C, 25°C, 30°C, and 35°C) with a shaking speed of 100 rpm. Digestion was carried out for various time intervals (2h, 3h, 4h, 5h, 6h). After digestion, 20 mL of 1.2 M KCl was added to each sample for further processing.

The enzymatic digestion mixture of *A. oxytropis* was filtered through fungal-specific filter cloth to separate the protoplasts from the remaining mycelia clumps. The protoplasts were then washed repeatedly with 1.2 M KCl to ensure complete collection. The resulting *A. oxytropis* protoplast suspension was centrifuged, and the supernatant was carefully removed. The protoplast pellet was resuspended in an appropriate volume of STC buffer and centrifuged again at 5,000 rpm for 15 min. After this step, the purified protoplasts were resuspended in STC buffer and counted using a hemocytometer. This process was repeated six times to accurately determine the protoplast concentration.

### 2.8 Protoplast transformation

Approximately 400 μL of prepared protoplasts (2–5 × 106 protoplasts/mL) was aliquoted into two sterile 1.5 mL centrifuge tubes. To each tube, 5 μg of silent vector was added respectively, mixed gently, and left to stand for 20 min (gently inverting the tubes). Then, 1 mL of 40% PTC Buffer was added to each tube, and the tubes were incubated at room temperature for 20 min. After that, 5 mL of TB3 Buffer (containing 50 μg/mL ampicillin) was added, and the protoplasts were cultured overnight at 30°C, shaking at 100 rpm.

The overnight-cultured *Alternaria oxytripis* protoplasts were centrifuged (5,000 rpm), and the supernatant was carefully removed. The protoplasts were then resuspended in an appropriate amount of regeneration lower layer media, followed by the addition of 1.5 μg/mL hygromycin and ampicillin. The mixture was quickly spread onto plates and cultured at 20°C for 24 h. Afterward, regeneration upper layer media containing 2.0 μg/mL hygromycin was poured onto the plates, and the culture continued at 20°C. Single colony transformants were observed on the plates. These transformants were picked and transferred onto hygromycin-resistant media for further selection. Wild-type *A. oxytropis* was used as a control. The transformants growing on the selective media were expanded for further experiments.

### 2.9 Screening of mutant strains (expression levels and swainsonine and intermediate product yield detection)

WT and the selected transformants were inoculated onto PDA media and cultured for 8 days. Fresh mycelia from each strain grown on the PDA media was collected, frozen in liquid nitrogen, ground into powder, and dried. The SW content and *swnR* gene expression level of the transformants were measured using the previously described methods, and silent strains (pSilent-*swnR*) with significantly reduced SW content and *swnR* gene expression were screened, three repetitions were set for each group. WT and the selected silent strains were inoculated on PDA media and continuously cultured for five generations. The SW content in the mycelia of the fifth generation was measured to determine whether there was a significant difference in SW synthesis between the silent strain and WT, and to assess whether the difference remained stable.

On the 8th day of culturing the fifth-generation wild-type and silent strains of *A. oxytropis*, mycelia and media from both WT and pSilent-*swnR* strains were collected. Using the previously described methods, LC-MS was used to detect the content of swainsonine (SW), pipecolic acid (Pa), and L-lysine (LY). The differences in the levels of these three products in the mycelia between the WT and pSilent-*swnR* strains were compared to determine the regulatory role of the swnR gene in the swainsonine biosynthesis pathway in *A. oxytropis*.

At the same time, total RNA from both WT and pSilent-*swnR* strains was extracted and reverse transcribed. Using cDNA as the template, the expression level of the *swnR* gene was detected via qRT-PCR to determine the effect of this gene's expression on swainsonine production.

### 2.10 Phenotypic verification of WT and silent strains

The wild-type and *swnR* gene silent strains of *A. oxytropis* were inoculated on PDA media and incubated upside down for 8 days in a fungal incubator at 20°C. The changes in colony morphology and the growth status of the strains were observed at various time points. And then mycelial slides were prepared; the mycelia were observed and recorded using an optical microscope. Finally, transmission electron microscopy (TEM) was used to observe and compare the ultrastructural differences of mycelia between WT and pSilent-*swnR* strains.

### 2.11 Metabolomics (sample processing, data analysis)

WT and the silent strains (pSilent-*swnR*) of *A. oxytropis* were inoculated on PDA media and cultured upside down in a fungal incubator at 20°C for 8 days. Mycelia from WT and two pSilent-*swnR* strains with the highest and lowest swainsonine production were collected. The mycelia of these three strains were divided into three groups, each with six samples. These samples were sent to Maiwei Company (Yangling, Shaanxi) for metabolomics analysis, with three replicates per group. Pairwise comparisons between the three groups were performed. Additionally, in-depth data mining was performed using the Maiwei Cloud platform (http://www.international.biocloud.net), enabling a series of personalized metabolomics operations, such as annotating differential metabolites using the KEGG database (Kanehisa and Goto, 2000) and enriching differential metabolic pathways.

### 2.12 Transcriptomics (sample processing, data analysis)

RNA sequencing was performed using the mycelia of WT and two pSilent-*swnR* strains. RNA extraction, quality verification, library preparation, and sequencing were performed by BMKCloud (Beijing, China). After sequencing, data analysis was conducted using the bioinformatics analysis pipeline provided by BMKCloud (http://www.biocloud.net). The Clean Data was assembled to obtain the Unigene library for this species. Based on this, the following analyses were performed: library quality assessment, structural level analysis, differential expression analysis, gene function annotation, and functional enrichment analysis. Furthermore, BMKCloud (http://www.biocloud.net) also provided options for in-depth data mining and various personalized transcriptomics operations, such as gene search and plotting, unique and shared gene analysis, protein interaction network construction, gene set enrichment analysis (GSEA), and co-expression analysis of differentially expressed genes.

### 2.13 Fungal inoculation

The technique for inoculating *Oxytropis glabra* plants with fungi (specific steps, based on Grum et al., 2012), and the cultivation conditions.

#### 2.13.1 Strain preparation

The silent strains 3-1 and 7-1 of *A. oxytropis* were inoculated on PDA media and cultured upside down in a fungal incubator at 20°C for 3 days.

#### 2.13.2 Seed preparation

Select intact *Oxytropis glabra* seeds and lightly sand the surface using 500-grit sandpaper to create slight surface damage. Sterilize the seed surface by rinsing the seeds in 75% ethanol for 30 s, followed by three rinses with sterile water. Then, soak the seeds in 10% sodium hypochlorite for 5 min, repeating this process three times, and finally rinse the seeds with sterile water three more times. The sterilized seeds are soaked in sterile water for 4 h.

Using sterile tweezers, remove the seed coat of the *Oxytropis glabra* seeds. The embryos without seed coats (only embryo, NC) are soaked in sterile water.

#### 2.13.3 Co-cultivated plants

Prepare the 3-day-old silent strains 3-1 and 7-1. Use a sterile pipette tip to punch small holes near the fungal colony, approximately 2 mm away from the colony edge. Carefully transfer the seed embryos onto filter paper to remove surface water, and then inoculate the radicle portion of the embryo into the small hole near the fungal colony, simulating the natural interaction between seeds and fungi. Place the co-cultivated samples in an artificial climate chamber and incubate upright under the following conditions: 20°C/16 h with level 3 light intensity, 18°C/8 h in darkness. After 5 days, transfer the growing seedlings to 230 mL culture bottles containing MS media, and continue culturing for another 14 days under the same conditions.

#### 2.13.4 Wild-type plants and NC plants

Wild-type plants (WTP): Select intact *Oxytropis glabra* seeds, sterilize and soak them in sterile water as described above, but do not remove the seed coat. Inoculate the seeds onto PDA media. After 5 days of growth, transfer the seedlings to 230 mL culture bottles containing MS media, and continue culturing for 14 days under the same conditions as described above.

OE plants: After sterilizing and removing the seed coat, inoculate the *Oxytropis glabra* embryos directly onto PDA media. After 5 days, transfer the seedlings to 230 mL culture bottles containing MS media, and continue culturing for 14 days under the same conditions as described above.

### 2.14 Detection of plant fungus colonization

#### 2.14.1 DNA extraction

DNA was extracted from freeze-dried preparations of pure cultures of fungus (~20 mg) grown on potato dextrose agar and from freeze-dried ground plant material (~20 mg) using the Plant/Seed DNA Mini Kit (Genstone Biotech). All extractions were performed according to the manufacturer's instructions. DNA was quantified with the Nanodrop One Ultra micro spectrophotometer (thermo scientific).

#### 2.14.2 Quantitative PCR

Using qPCR to detect fungal endophyte colonization in plants. The method described in (Cook et al., 2009). Using a CHROMO 4 quantitative PCR detector (Bio-Rad Laboratories Inc., Hercules, CA). The limit of quantitation was 0.2 pg endophyte DNA per ng total DNA. The primers used were ITS 5 (5′ GGA AGT AAA AGT CGT AAC AAG G 3′) and OR1a (5′ GTC AAA AGT TGA AAA TGT GGC TTG G 3′), which amplify the ITS region of the rDNA region (Cook et al., 2009). Primers designed by Tsingke.

### 2.15 Scanning electron microscopy (SEM)

The stems and leaf tissues of the 14-day-old wild-type, OE, and co-cultivated plants are taken from the culture bottles. These tissues are prepared for scanning electron microscopy (SEM) to observe the colonization of fungal mycelia in the plants for each group.

### 2.16 Phenotypic observation of WT, silent, and NC groups (OE group)

After 14 days of growth, wild-type, OE, and co-cultivated plants are taken out of the culture bottles. The growth morphology of the plants is observed, and the total height, number of leaves, number of branches, and fresh weight of the seedlings are recorded.

### 2.17 Metabolite detection of WT, silent, and NC groups (SW and intermediate product yield detection)

After *Oxytropis glabra* seeds were cultured on PDA media for 5 days, they were transferred to culture bottles containing MS media and cultured for 14 days. The seedlings from each group were collected.

Swainsonine extraction method: weigh 25 mg of dried plant sample into a 2 mL centrifuge tube using an analytical balance. Grind the sample into a fine powder using a grinder. Add 1.5 mL of anhydrous ethanol and shake for 18 h to extract. After centrifugation, collect the supernatant. The sediment can be re-extracted using the same method to increase extraction efficiency. Combine the supernatants and filter through a microporous membrane into a 1 mL auto-sampler vial. The swainsonine content in the extract is detected using LC-MS, under the same conditions and calculation methods as used for swainsonine detection in fungal samples.

The swainsonine content in the extract is divided by the dry weight of the seedlings to obtain the swainsonine content per milligram of plant seedlings.

### 2.18 Statistical analysis

The analyses not explicitly stated in this study were all conducted using IBM SPSS Statistics software (version 26.0). A two-factor correlation analysis (Spearman) was employed to assess the relationship between the production of swainsonine (SW) and the expressions of genes *swnH1, swnH2, swnN, swnK*, and *swnR*. Subsequently, the correlations of the genes were analyzed using Origin software. One-way analysis of variance (ANOVA) was performed on the expression levels of genes within the *SWN* gene cluster, the contents of SW, Pa, and LY detected by LC-MS, as well as various statistical data such as plant fungal biomass, fresh weight, height, leaf number, and branch number. The significance threshold was as follows: ^****^P < 0.0001 indicates an extremely significant difference between groups (except for the three silent transformant mycelia where the content of SW was not reached *P* < 0.0001 compared with the wild type, so the *P* values used were *P* < 0.05 and *P* < 0.01, while for the remaining experiments, *P* < 0.0001 was used).

## 3 Results

### 3.1 Correlation analysis between *SWN* gene cluster and SW yield

**Figure 2:**
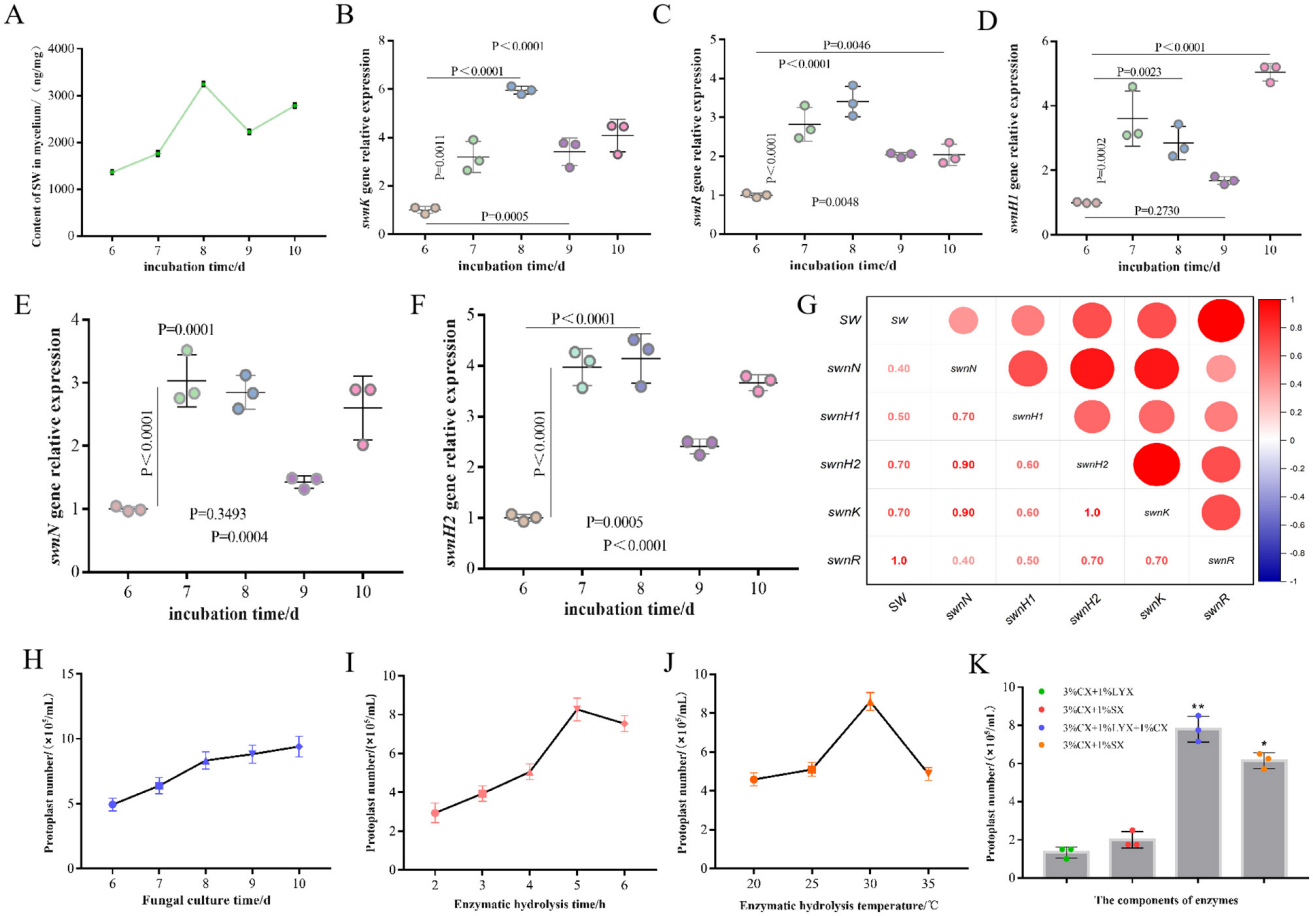
The *swnR* gene is a key catalytic enzyme gene in the swainsonine biosynthesis pathway of *Alternaria oxytropis*. **(A)** Swainsonine yield of *A. oxytropis* cultured from day 6 to day 10. **(B–F)** Expression levels of *swnK, swnR, swnH1, swnN*, and *swnH2* genes genes in *A. oxytropis* cultured from day 6 to day 10. **(G)** Correlation analysis between swainsonine production and the expression levels of genes in the SWN gene cluster in *A. oxytropis*. **(H)** Effect of culture age on the number of *A. oxytropis* protoplasts prepared. **(I)** Effect of different enzymatic digestion durations on the number of *A. oxytropis* protoplasts prepared. **(J)** Effect of different enzymatic digestion temperatures on the number of *A. oxytropis* protoplasts prepared. **(K)** Effect of different enzyme components on the number of *A.oxytropis* prepared protoplasts. ^*^P < 0.05 indicates a significant difference between groups. ^**^P < 0.01 indicates an extremely significant difference between groups.

In order to screen and identify key genes involved in swainsonine biosynthesis in *A. oxytropis*, we measured the swainsonine content in the fungal mycelia from days 6 to 10, along with the expression levels of five genes from the SWN gene cluster (Figures 2B–F). We found that the expression levels of *swnH2, swnK*, and *swnR* peaked on day 8 during the observation period, which closely mirrored the trend in swainsonine production (Figures 2A–C, F). Correlation analysis further demonstrated a strong positive correlation (*R* = 1.000) between swainsonine production and *swnR* gene expression in *A. oxytropis* (Figure 2G). These results indicate that the *swnR* gene is a key factor involved in the swainsonine biosynthesis pathway in *A. oxytropis*.

### 3.2 Effect of *swnR* gene expression on SW production

To perform gene editing on *A. oxytropis*, it was crucial to obtain a sufficient quantity of protoplasts for subsequent experimental procedures. Thus, we undertook a series of explorations and optimizations for protoplast preparation conditions. By treating 8-day-old mycelia with different enzyme mixtures and durations, we found that a mixture of three enzymes (3% CX+1% LYX+1% CX) produced the highest number of protoplasts, approximately 8.3 × 105 protoplasts/mL, after 5 h of treatment (Figures 2H, I, K). Beyond this point, protoplast lysis began to occur which reduced the number of usable protoplasts (Figures 2I, K). We also optimized the enzymatic digestion temperature and fungal age for protoplast preparation. Results showed that the enzymatic solution was most effective at 30°C, producing the highest yield of protoplasts. As the temperature increased, protoplast yield significantly decreased (Figure 2J). We found 8-day-old mycelia consistently yielded sufficient protoplasts therefore 8d was used for subsequent experiments (Figure 2H).

Before conducting transformation experiments on *A. oxytropis*, it was essential to construct a silencing vector targeting the *swnR* gene. As part of this process, antibiotic sensitivity testing was performed to determine the appropriate type and concentration of antibiotics for selecting transformants and preparing selective media for protoplast transformation. Previous laboratory research suggested that a specific concentration of hygromycin could inhibit the growth of *A. oxytropis*. To further verify the fungal sensitivity to hygromycin, *A. oxytropis* was inoculated onto media containing varying concentrations of hygromycin. Results indicated that a concentration of 2.5 μg/mL completely inhibited the growth of *A. oxytropis* (Supplementary Figure S1A).

Given the sensitivity of *A. oxytropis* to hygromycin, the pSilent-1 vector containing the hygromycin resistance gene was selected as the empty vector for gene silencing. The pSilent-*swnR* silencing construct had been successfully constructed as confirmed by complete plasmid construct sequencing (Supplementary Figure S1B). The pSilent-*swnR* vector was then introduced into *A. oxytropis* protoplasts via PEG-mediated transformation. After 10–14 days, colonies appeared on PDA plates containing hygromycin. Four hygromycin-resistant transformants were randomly selected, and of these, three were confirmed to harbor the pSilent-*swnR* vector (Supplementary Figure S1C). These confirmed colonies were subsequently transferred to PDA plates for further analysis.

**Figure 3:**
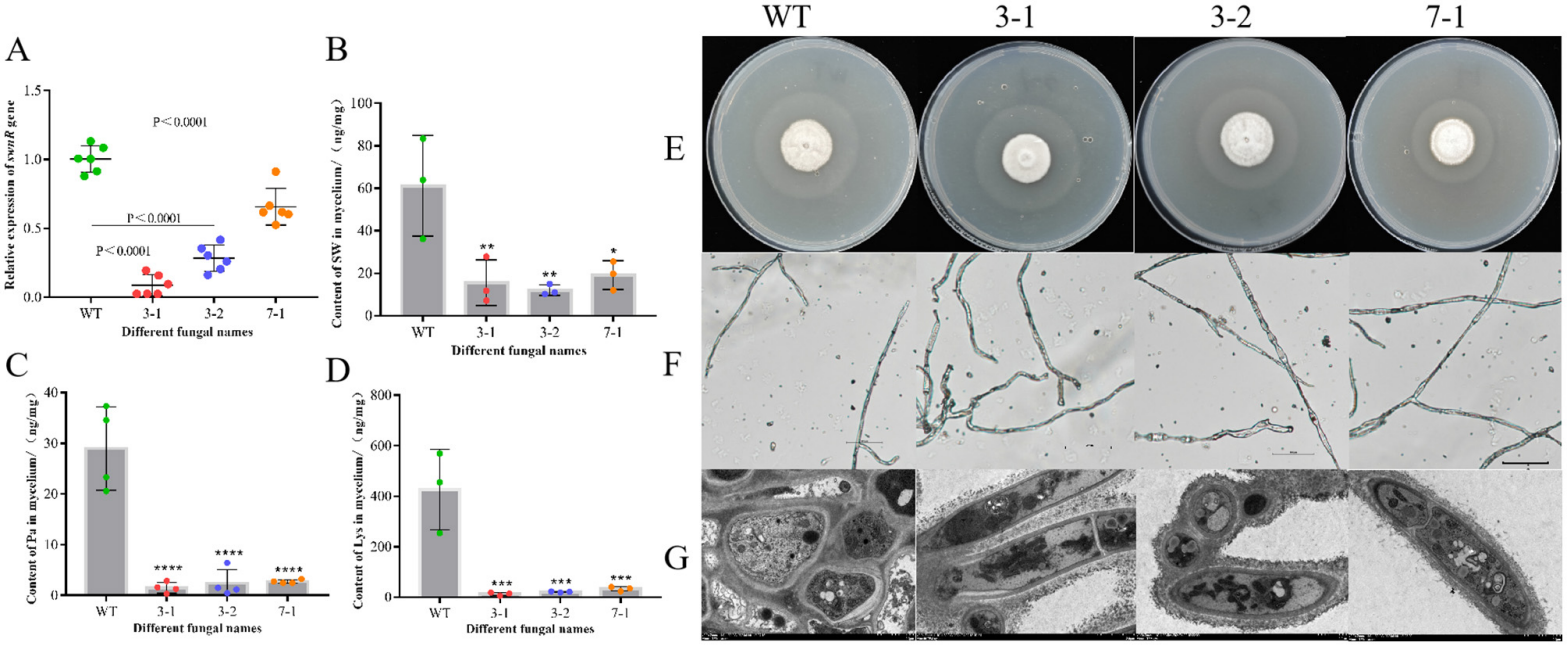
Silencing of the *swnR* gene reduces swainsonine production in *Alternaria oxytropis*. **(A)** Expression levels of the *swnR* gene in wild-type (WT) and silenced strains 3-1, 3-2, and 7-1. **(B)** Swainsonine content in the mycelia of WT and silenced strains 3-1, 3-2, and 7-1. **(C)** Pa content in the mycelia of WT and silenced strains 3-1, 3-2, and 7-1. **(D)** Lys content in the mycelia of WT and silenced strains 3-1, 3-2, and 7-1. **(E–G)** Colony morphology of wild-type and silenced strains on PDA media and the morphological characteristics of their mycelia under optical and transmission electron microscopes. ^*^P < 0.05, ^**^P < 0.01, ^***^P < 0.001, ^****^P < 0.0001.

The expression levels of the *swnR* gene in the wild-type (WT) strain and the WT strain transformed with the empty vector pSilent-1 were comparable as determined by qPCR quantification. The mRNA levels of *swnR* were significantly reduced in three of the transformants (3-1, 3-2, 7-1, 91.30%, 71.61%, 34.32%, respectively), with strain 3-1 exhibiting a 91.51% reduction in *swnR* gene expression relative to the wild-type (*P* < 0.0001) (Figure 3A). Microscopic and ultrastructural observations of colony mycelia from the wild-type *A. oxytropis* (WT) and the silenced strains 3-1, 3-2, and 7-1 revealed no notable differences (Figures 3E–G).

Silencing the *swnR* gene led to a significant reduction in swainsonine production in *A. oxytropis*. Since the culture media used for growing *A. oxytropis* is plant-based and already contains the precursor lysine and the intermediate product L-pipecolic acid, we were only able to assess the levels of swainsonine and related compounds in the mycelia. As illustrated in Figure 3B, swainsonine levels in the mycelia of all three transformants were significantly reduced (*P* < 0.05), with strains 3-1 and 3-2 showing highly significant reductions in swainsonine content (*P* < 0.01). Figures 3C, D further indicate that lysine and pipecolic acid levels in the mycelia of all three transformants were also significantly decreased (*P* < 0.0001). These findings suggest that the reduction in *swnR* gene expression directly limits the availability of precursors for the swainsonine biosynthesis pathway, thereby fundamentally decreasing swainsonine production.

### 3.3 Effects of *swnR* gene expression on transcriptome and metabolome of *A. oxytropis*

**Figure 4:**
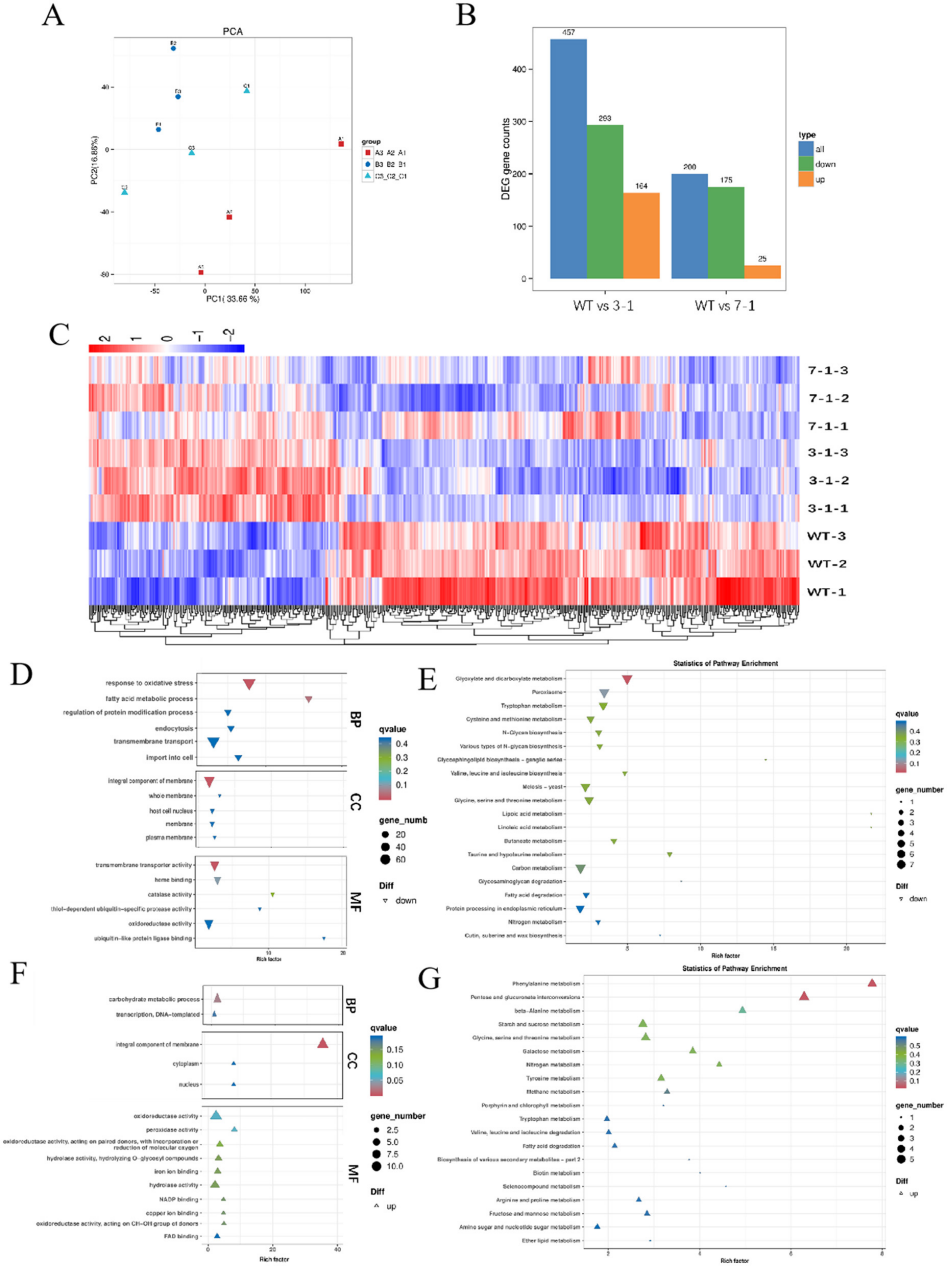
Transcriptomic analysis of WT and silenced strains 3-1 and 7-1. **(A)** PCA score graphs of all samples [**(A–C)** represent WT, 3-1, and 7-1]. **(B)** DEG genes counts. **(C)** The Heatmap of the DEGs. **(D, E)** Enrichment analysis of down-regulated differentially expressed genes GO and KEGG in silenced strain 3-1 vs WT. **(F, G)** Enrichment analysis of up-regulated differentially expressed genes GO and KEGG in Silenced strain 3-1 vs. WT.

Further investigation was conducted to assess the effects of varying degrees of *swnR* gene silencing on fungal growth, development, and the synthesis and secretion of secondary metabolites. Principal component analysis (PCA) score plots revealed distinct separations among the silenced strains 3-1, 7-1, and WT (Figure 4A), with the separation trends correlating with changes in *swnR* gene expression levels. Compared with WT, strain 3-1 exhibited 164 upregulated genes and 293 downregulated genes, while strain 7-1 exhibited 25 upregulated and 175 downregulated genes. Cluster analysis further highlighted significant differences between the wild-type and each of the silenced strains (Figures 4B, C). Gene Ontology (GO) enrichment analysis of the upregulated genes in strain 3-1 revealed that these genes were predominantly involved in multiple metabolic pathways, including the carbohydrate metabolic process, oxidoreductase activity, and transmembrane transporter activity, indicating roles in secondary metabolism networks, cellular structural adaptations, and metabolic homeostasis mechanisms (Figure 4F). KEGG pathway enrichment analysis demonstrated that downregulation of *swnR* significantly activated several primary metabolic pathways in *Alternaria oxytropis*, including phenylalanine metabolism, carbohydrate metabolism, and porphyrin and chlorophyll metabolism. Notably, there was a marked upregulation in pathways associated with the metabolism of aromatic compounds and other secondary metabolites (Figure 4G). Conversely, downregulated genes in strain 3-1 were mainly enriched in pathways related to oxidative stress responses, substance transport, membrane dynamics, and transmembrane transporter activity, as revealed by GO analysis (Figure 4D). KEGG analysis further indicated significant suppression of several key metabolic pathways, including glyoxylate and dicarboxylate metabolism, peroxisome activity, and lipid metabolism and degradation (Figure 4E). In contrast, upregulated genes in strain 7-1 were enriched in only three metabolic categories: phenylalanine metabolism, transmembrane transporter activity, and membrane components. The downregulated genes in strain 7-1 showed patterns similar to those observed in strain 3-1, involving oxidative stress responses, carbon metabolism, glyoxylate and dicarboxylate metabolism, and membrane dynamics, with only a few biological pathways—characterized by low enrichment factors and limited statistical significance—differing between the two groups (Supplementary Figures S2A–F).

Transcriptomic analysis of swainsonine biosynthesis-associated genes revealed significant differential expression. Comparative assessment of expression levels within the *SWN* gene cluster and the P5CR gene between the wild-type strain and the silenced strain demonstrated significantly reduced expression of *swnR* (TRINITY_DN2817_c1_g1), *swnN* (TRINITY_DN2817_c0_g1), *swnK* (TRINITY_DN7706_c0_g1), and *P5CR* (TRINITY_DN1901_c0_g1). Furthermore, both coding sequence (CDS) and amino acid sequence analyses indicated that *swnH1* and *swnH2 c*orrespond to the same genetic locus (TRINITY_DN8537_c0_g1). This gene (*swnH1*/*H2*) similarly exhibited significantly reduced expression (Supplementary Table S4).

**Figure 5:**
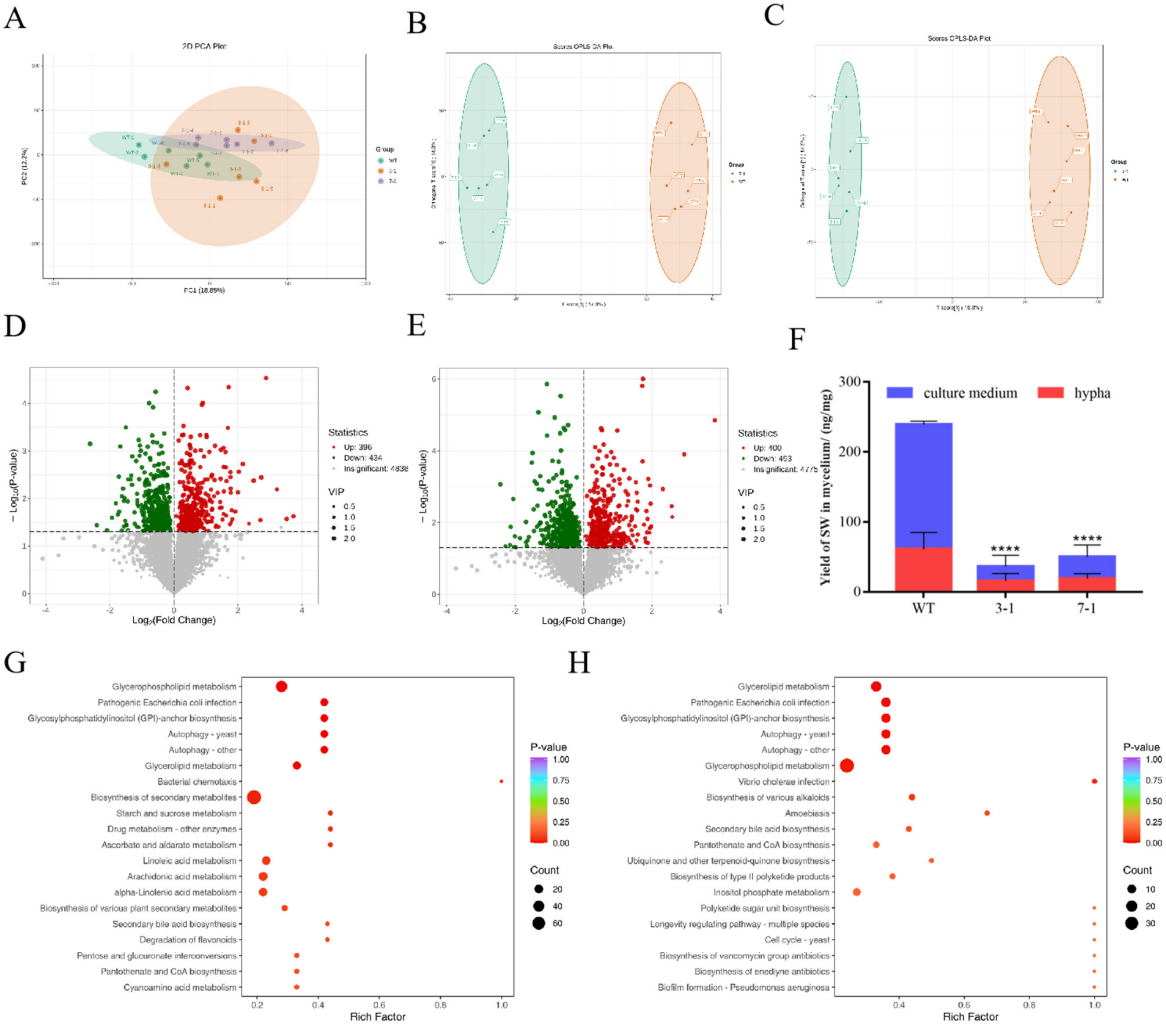
Metabolomic analysis of WT and silenced strains 3-1 and 7-1. **(A)** PCA score graphs of all samples. **(B)** OPLS-DA_scorePlot between silenced strain 3-1 and WT. **(C)** OPLS-DA_scorePlot between silenced strain 7-1 and WT. **(D)** Volcano map between silenced strain 3-1 and WT. **(E)** Volcano map between silenced strain 7-1 and WT. **(F)** The content distribution of SW in mycelia and culture media of wild-type strains, silenced strains 3-1 and 7-1. **(G)** KEGG enrichment analysis for differential metabolites between silenced strains WT and 3-1. **(H)** KEGG enrichment analysis for differential metabolites between silenced strains WT and 7-1.

After identifying significant transcriptomic differences among the silenced strains 3-1, 7-1, and WT, we further explored the metabolic differences between the wild-type and the two silenced strains by integrating transcriptomic and metabolomic analyses. Non-targeted metabolomics was employed to detect metabolites in each group, aiming to investigate the metabolic response mechanisms triggered by changes in *swnR* gene expression levels. Across the three groups, a total of 6,841 metabolites were identified, with the most abundant classes including amino acids and their derivatives, benzene and substituted derivatives, heterocyclic compounds, and organic acids and their derivatives (Figures 5D, E). PCA score plots revealed that while there was some overlap between the metabolites of silenced strains 3-1, 7-1, and WT, clear separations were also evident (Figure 5A). PLS-DA analysis further confirmed distinct separations between the silenced strains and the wild-type, indicating that changes in *swnR* expression significantly impact the metabolic profiles of *A. oxytropis* (Figures 5B, C). Specifically, 830 differential metabolites were identified between the wild-type and strain 3-1, with 396 metabolites significantly upregulated and 434 downregulated (Figure 5D). These metabolites were mainly enriched in pathways related to carbon metabolism, lipid metabolism, antioxidative responses, autophagy and organelle renewal, and secondary metabolite biosynthesis, showing high consistency with transcriptomic results (Figure 5G, Supplementary Figure S3). Similarly, 893 differential metabolites were found between the wild-type and strain 7-1, with 400 significantly upregulated and 493 downregulated (Figure 5E). As in strain 3-1, these differential metabolites were enriched in pathways related to secondary metabolite biosynthesis, lipid metabolism, and amino acid metabolism. However, a notable distinction in the strain 7-1 group was the enrichment of differential metabolites in the pathway “microbial metabolism in diverse environments,” suggesting that microbial metabolic activities were strongly regulated (Figure 5H, Supplementary Figure S3B). These findings underscore the critical role of *swnR* in regulating secondary metabolism pathways, suggesting that modulation of *swnR* expression profoundly impacts the production of bioactive metabolites and the adaptive capacity of *A. oxytropis* under environmental stress.

Metabolomic analysis identified alterations in metabolites associated with swainsonine biosynthesis. Specifically, decreased abundance of L-pipecolic acid and L-Saccharopine was observed. These metabolites were co-enriched within the same metabolic pathway. In contrast, levels of L-lysine, L-glutamic acid, and L-proline showed no significant differences (Supplementary Table S5). Other putative biosynthetic intermediates were not detected in our metabolomics dataset.

To test whether changes in *swnR* gene expression affect material transport across the cell membrane in *A. oxytropis*, we used LC-MS to measure swainsonine levels in both the mycelia and culture media of WT and the 3-1 and 7-1 silenced strains on the 8th day. In WT, the ratio of swainsonine content in the mycelia to the culture media was 1:3, while in the 3-1 and 7-1 strains, the ratios were ~ 1:1 and 1:1.5, respectively (*P* < 0.0001) (Figure 5F).

### 3.4 Changes in *swnR* gene expression affect swainsonine production in symbiotic plants

**Figure 6:**
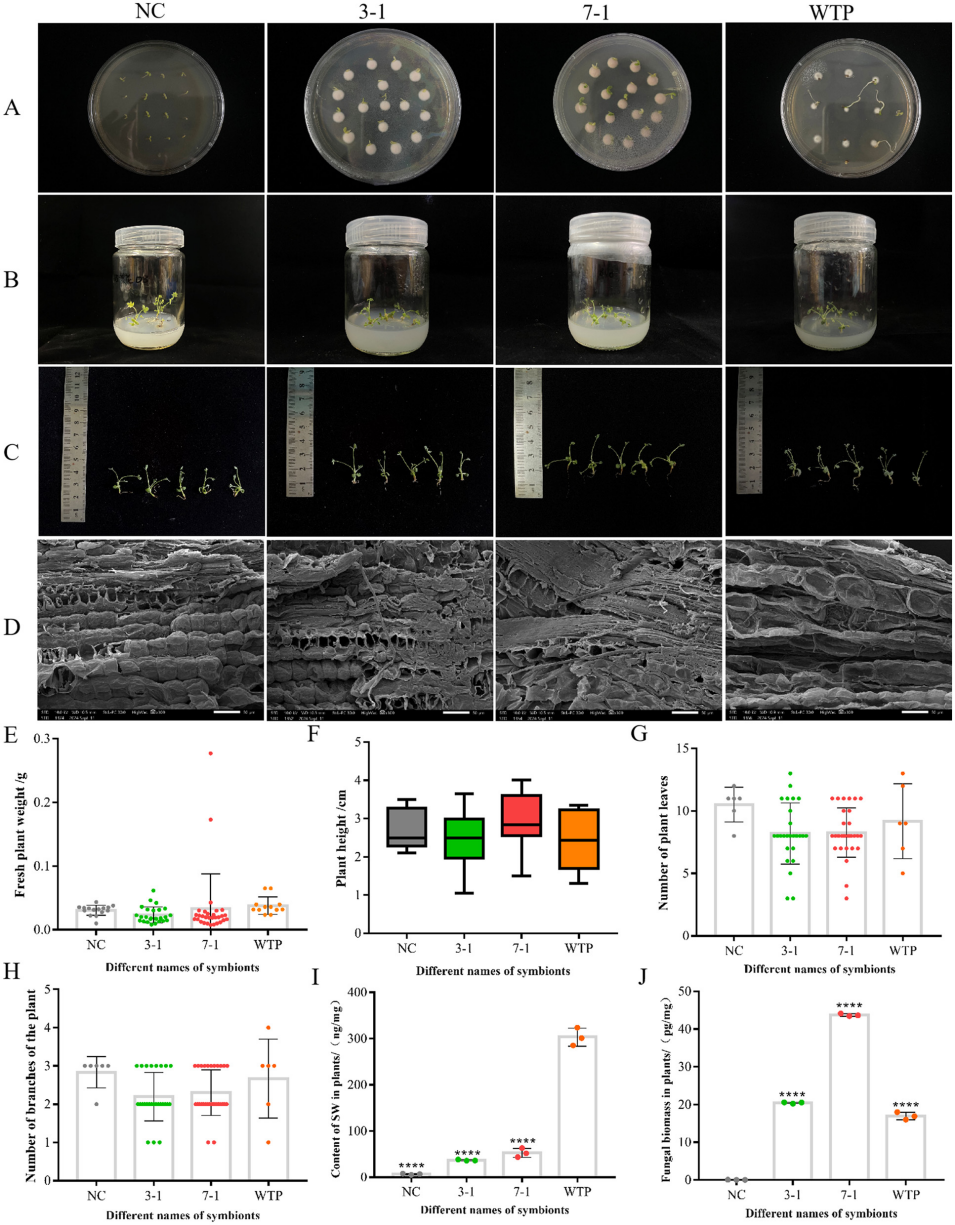
Symbiosis with silenced strains reduces swainsonine content in *Oxytropis* plants. **(A, B)** Morphological observation of each group of plant seedlings in PDA media and MS media. **(C)** The visual morphology of each group of plant seedlings. **(D)** Scanning electron microscopy images of plant seedlings from each group, showing longitudinal sections of petioles. **(E–H)** Fresh weight, plant height, leaf number, and branch number of seedlings in each group. **(I)** Swainsonine content in seedlings from each group. **(J)** Fungal biomass in seedlings from each group. ^****^P < 0.0001 indicates an extremely significant difference between groups.

To investigate whether the silenced *A. oxytropis* strains affect the growth of *Oxytropis glabra* seedlings, we co-cultivated the silenced strains with peeled seed embryos. For unpeeled *O. glabra* seeds (WTP), fungal mycelia appeared around the seedlings 5 days after germination on PDA media and continued to grow around the seedlings after they were transferred to MS media (Figures 6A, B). In contrast, no fungal mycelia was observed around the peeled seed embryos under the same conditions. For seedlings co-cultivated with the silenced strains 3-1 and 7-1, no fungal mycelia initially appeared on the PDA media, but 14 days after transferring to MS media, fungal growth became visible around the roots (Figure 6C). Further analysis using scanning electron microscopy revealed fungal mycelia from both the silenced and wild-type strains within longitudinal sections of petioles from *O. glabra* seedlings. The mycelia extended into the intercellular spaces between the cell walls, exhibiting branching. However, in the longitudinal sections of petioles from plants grown from peeled seed embryos (NC), no fungal mycelia was detected (Figure 6D).

We observed, measured, and analyzed the fresh weight, plant height, leaf number, and branching number of *Oxytropis glabra* seedlings co-cultivated with WTP as well as the silenced strains 3-1 and 7-1. The statistical analysis revealed no significant differences in these growth parameters between seedlings co-cultivated with the silenced strains and those co-cultivated with WTP (Figures 6E–H). Furthermore, comparisons of fresh weight and plant height between seedlings from peeled and unpeeled seeds showed that the presence or absence of fungal symbiosis had no significant effect on these growth parameters. Therefore, it can be inferred that the silenced strains 3-1 and 7-1 do not have a significant negative impact on the growth and development of *O. glabra* seedlings. There were subtle observable differences between WT and silenced strains, however, these differences were not statistically significant.

After measuring the swainsonine content in the fungal mycelia and culture media, we observed that swainsonine levels in the silenced strains 3-1 and 7-1 decreased to varying degrees compared to WT. To determine if co-cultivation with these silenced strains would result in similar reductions in *Oxytropis glabra*, we set up negative controls (NC) and positive controls (WTP) and used LC-MS to measure the swainsonine content in the seedlings. The results showed that seedlings from peeled seeds contained the lowest levels of swainsonine, while unpeeled wild-type seedlings had the highest levels. Seedlings co-cultivated with the silenced strains 3-1 and 7-1 exhibited a significant reduction in swainsonine content compared to the wild-type (*P* < 0.0001) (Figure 6I). Additionally, the swainsonine content in seedlings co-cultivated with strain 7-1 was slightly higher than that in seedlings co-cultivated with strain 3-1, consistent with the observed changes in swainsonine levels in the fungal mycelia and culture media.

The detection of swainsonine content in the silenced symbionts was consistent with our initial experimental expectations. To more precisely compare the impact of the silenced strains on swainsonine levels in the symbiotic plants, we also measured the fungal biomass within the symbionts. The results showed that the biomass of the 7-1 group was significantly higher than that of the 3-1 group, nearly double (Figure 6J). This suggests that the silenced strain 7-1 has a higher infection efficiency and a greater ability to establish symbiosis with the plant. However, based on the ratio of swainsonine production by the strains, the swainsonine content in plants co-cultivated with strain 7-1 was about three times that of strain 3-1 group. The actual results, however, did not align with this prediction, the reason for this discrepancy remains to be further investigated. This divergence could be attributed to unknown regulatory mechanisms that influence swainsonine production during the symbiotic interaction between the fungus and the plant.

## 4 Discussion

Previous studies have shown that knocking out the *swnR* gene in *Metarhizium robertsii* (Luo et al., 2020) can reduce swainsonine production in fungi by approximately fivefold, suggesting that this gene plays a key role in the primary swainsonine biosynthesis pathway in *M. robertsii* (Luo et al., 2020). Similarly, Sun et al. (2021) knocked out the *swnR* gene in *M. anisopliae* and observed a significant reduction in swainsonine production, although swainsonine was still produced. Furthermore, disruption of the gene caused cell wall damage in *M. anisopliae*. Before conducting *swnR* gene silencing in *A. oxytropis*, we attempted to knock out the *swnR* gene, but we were unable to obtain any viable knockout transformants (data not shown). Meanwhile, Das et al. silenced the *swnT* gene in *S. leguminicola* and found that as silencing efficiency increased, the growth of the fungus was clearly inhibited. However, the maximum silencing efficiency was only 85%, and they were unable to obtain fully silenced strains (Das et al., 2023). We speculate that the *swnR* gene may behave similarly—complete loss of expression may not be compatible with the survival of *A. oxytropis*, which is why we opted for gene knockdown rather than total knockout. Subsequent comparison of the metabolomic results between the silenced strains and the wild-type revealed that reduced *swnR* gene expression led to the enrichment of some differential metabolites in the autophagy as shown in yeast pathway. This suggests that a significant reduction in *swnR* gene expression might trigger autophagy in *A. oxytropis* cells, potentially leading to cell death. This could explain why we were unable to obtain total *swnR* knockout strains in our earlier experiments. However, this hypothesis requires further experimental validation.

Comprehensive analysis of GO and KEGG enrichment data from transcriptomic profiling in this study provides important insights into the regulatory role of *swnR* in the biosynthesis of swainsonine in *A. oxytropis*. Differentially expressed genes (DEGs) were significantly enriched in aromatic amino acid metabolism pathways, including phenylalanine, tyrosine, and tryptophan metabolism, suggesting that *swnR* may contribute to the supply of precursor materials essential for swainsonine biosynthesis, which originates from nitrogen-containing amino acid metabolic pathways. These pathways have previously been implicated in the production of alkaloids and other specialized metabolites (Cook et al., 2014), and may thus represent critical metabolic nodes regulated by *swnR*. The concurrent enrichment of DEGs in carbohydrate metabolism pathways, such as starch and sucrose metabolism and galactose metabolism, further indicates potential alterations in energy utilization and carbon skeleton distribution upon *swnR* downregulation. This metabolic shift likely reflects a compensatory response aimed at maintaining cellular energy balance when secondary metabolite biosynthesis is perturbed. Moreover, the enrichment of GO terms related to oxidoreductase activity and metal ion binding suggests that *swnR* may directly or indirectly regulate a suite of oxidoreductases, potentially playing a pivotal role in structural modifications during swainsonine biosynthesis. Finally, the significant enrichment of DEGs associated with extracellular regions and membrane components indicates that *swnR* may also influence the transport and secretion of swainsonine or its intermediates, thereby further modulating its accumulation. Together, these findings demonstrate that *swnR* exerts multifaceted control over swainsonine biosynthesis and mobilization by coordinating precursor metabolism, enzymatic modification systems, and membrane-associated processes.

Comparative transcriptomic analysis of the wild-type (WT) and *swnR*-silenced strains demonstrated significant downregulation of key SW biosynthetic genes (*swnK, swnN, swnH2*, and *swnH1*), with the remaining genes in the *SWN* cluster being downstream of *swnR* and subject to its regulatory control. The reduced expression of *swnR* likely impairs L-pipecolic acid (Pa) production, thereby disrupting saponin biosynthesis and leading to the observed downregulation of the *SWN* gene cluster. Notably, transcriptomic data also revealed decreased expression of *P5CR*, corroborating findings by Yang et al. (2025), who reported diminished *swnR* expression upon *P5CR* knockout. These results collectively support the hypothesis of Lu et al. (2016) that *P5CR* contributes to Pa synthesis via Δ^2^-piperideine-6-carboxylate (P6C) in the saponin biosynthetic pathway.

Metabolomic profiling identified five SW-related metabolites, among which L-saccharopine and Pa levels were reduced, while L-lysine remained unchanged. This discrepancy with LC-MS data may arise from the presence of these metabolites in both monomeric and complex structural forms in metabolomic databases, complicating direct quantification. Integrating metabolomic and LC-MS analyses, the decline in lysine, L-saccharopine, and Pa levels aligns with reduced *swnR* expression, indicating that *swnR* not only participates in the process of P6C converting to Pa, but may also indirectly regulate the accumulation of precursor pathways and intermediate products. However, no significant changes in L-glutamic acid or L-proline were detected, indicating that *swnR* and *P5CR* exhibit only partial functional overlap and do not directly regulate P5C-derived L-proline synthesis or P5C-to-glutamate conversion.

Limitations in metabolomic detection were evident, as critical intermediates such as 6-aminooctanedioic acid, Δ^2^-piperideine-2-carboxylate (P2C), P6C, 1-oxo-indolizine, and 1-hydroxy-indolizine were not detected—likely due to low abundance, instability, or the absence of reference standards. These gaps highlight the need for advanced analytical methodologies to fully elucidate the SW biosynthetic pathway.

For many years, swainsonine was thought to be a plant-derived secondary metabolite. However, recent studies have shown that *A. oxytropis*, an endophyte symbiotic with locoweed, is the actual source of swainsonine. Cook's research demonstrated that *Alternaria* spp. primarily reside in the seed coat, and plants grown from seed embryos with the seed coat removed contain very little (0.0003 ± 0.00009%) or no swainsonine (Grum et al., 2012). In this study, seedlings grown from coat -peeled seed embryos also contained trace amounts of swainsonine, at 0.00667%, which is more than 20 times higher than the amount reported by Cook. This discrepancy may be due to the differences in locoweed species; however, the ability of *Oxytropis glabra* to produce swainsonine independently has not been further studied. The swainsonine content in locoweed is influenced by various factors, including internal factors such as the plant host and its endophytic fungi, as well as external environmental conditions. It has been reported that, compared to controls, the swainsonine concentration in *O. sericea* and *A. oxytropis* plants grown under drought conditions was significantly higher (Oldrup et al., 2010). Based on the observed growth of the seedlings and the measured swainsonine content, we found that while the silenced strains 3-1 and 7-1 exerted a limited impact on seedling growth, they still subtly retarded seedling development. However, the reduction in swainsonine content was far more pronounced than the negative effects on growth. Species within the genera *Astragalus* and *Oxytropis*, which belong to the legume family, are well-known for forming symbiotic relationships with nitrogen-fixing bacteria, thereby influencing the plant's nitrogen status (Valdez Barillas et al., 2007). Previous studies have shown that, irrespective of the average swainsonine concentration, all species exhibit a positive response to nitrogen in terms of biomass, leaf photosynthesis, and pigment production (Delaney et al., 2011). Moreover, other experiments have demonstrated that swainsonine concentrations in *O. sericea* or *A. oxytropis* do not differ when grown under either optimal or nitrogen-deficient conditions (Oldrup et al., 2010). In light of our experimental results, it can be inferred that co-cultivation with the silenced strains significantly reduced swainsonine content in the seedlings, while having a relatively minimal impact on the overall growth and development of *O. glabra* seedlings, suggesting great potential of this approach for remediation of the remediation of locoweed problem or even turn toxic plants into nutritious feed.

## 5 Conclusions

This study demonstrates that the transcription factor *swnR* serves as a master regulator of swainsonine (SW) biosynthesis in *Alternaria oxytropis*. Silencing *swnR* significantly downregulated the entire *SWN* gene cluster and disrupted SW production. Crucially, we firstly established that the strategy targeting *swnR* can effectively reduce the content of swainsonine in locoweed, while also maintaining the endophytic symbiotic relationship of fungi, providing a novel biological control strategy for preventing fungal poisoning in livestock.

## Data Availability

The original data presented in this study are included in the article/Supplementary material. If you have any further questions, please contact the corresponding author.
