# Supplementary material for: siRNA-Silencing of swnR gene greatly reduced biosynthesis of swainsonine in Alternaria oxytropis without affecting the growth characteristics of leguminous host
Source: Front Microbiol. 2025 Aug 25;16:1641192. doi: 10.3389/fmicb.2025.1641192 (PMC12415035; doi:10.3389/fmicb.2025.1641192)
Supplement: Supplementary file 1 [file Data_Sheet_1.PDF]

Supplementary Material

siRNA-Silencing of *swnR* gene greatly reduced biosynthesis of swainsonine in *Alternaria oxytropis* without affecting the growth characteristics of leguminous host

Yu Zhang, Liwen Yang, Yange Li, Shiyu Tang,Yiqingqing Zhang, Pinzhi Sun, Hao Lu*

College of Veterinary Medicine, Northwest A&F University, Yangling 712100, Shaanxi, China

*** Correspondence:**
Corresponding Author: Hao Lu
luhao@nwsuaf.edu.cn

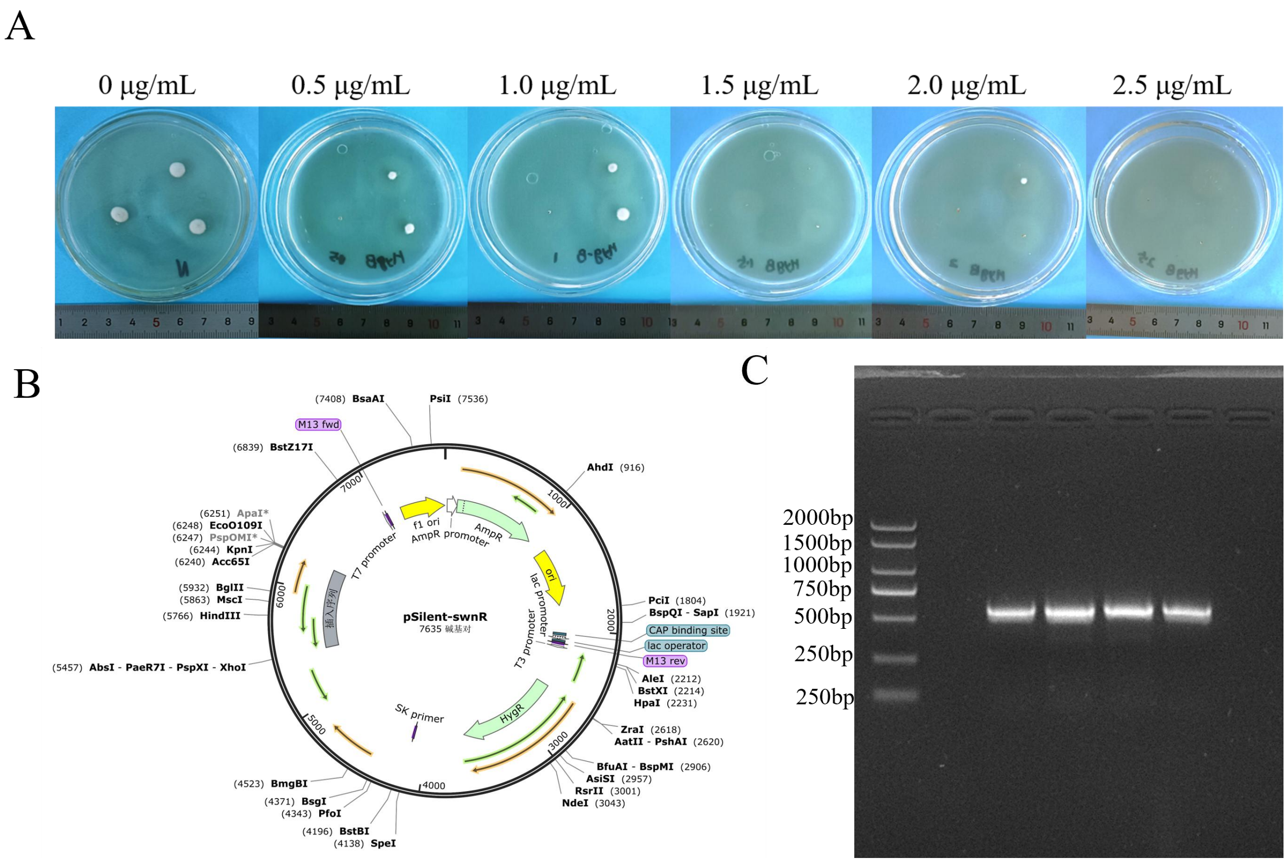

1 Supplementary Figures

FIG S1 Silent vector construction and transformation screening**. (A)** Effects of different concentrations of hygromycin B on the growth of *A. oxytropis*. **(B)** pSilent-swnR vector detection map. **(C)**The specific fragment of swnR gene silencing strain of *A. oxytropis* (Lane 1: Wild type strain of *A. oxytropis*; Lane 2: pSilent-*swnR* vector; Lane 3-5: The specific fragment of *swnR* gene silencing strain of *A. oxytropis*).

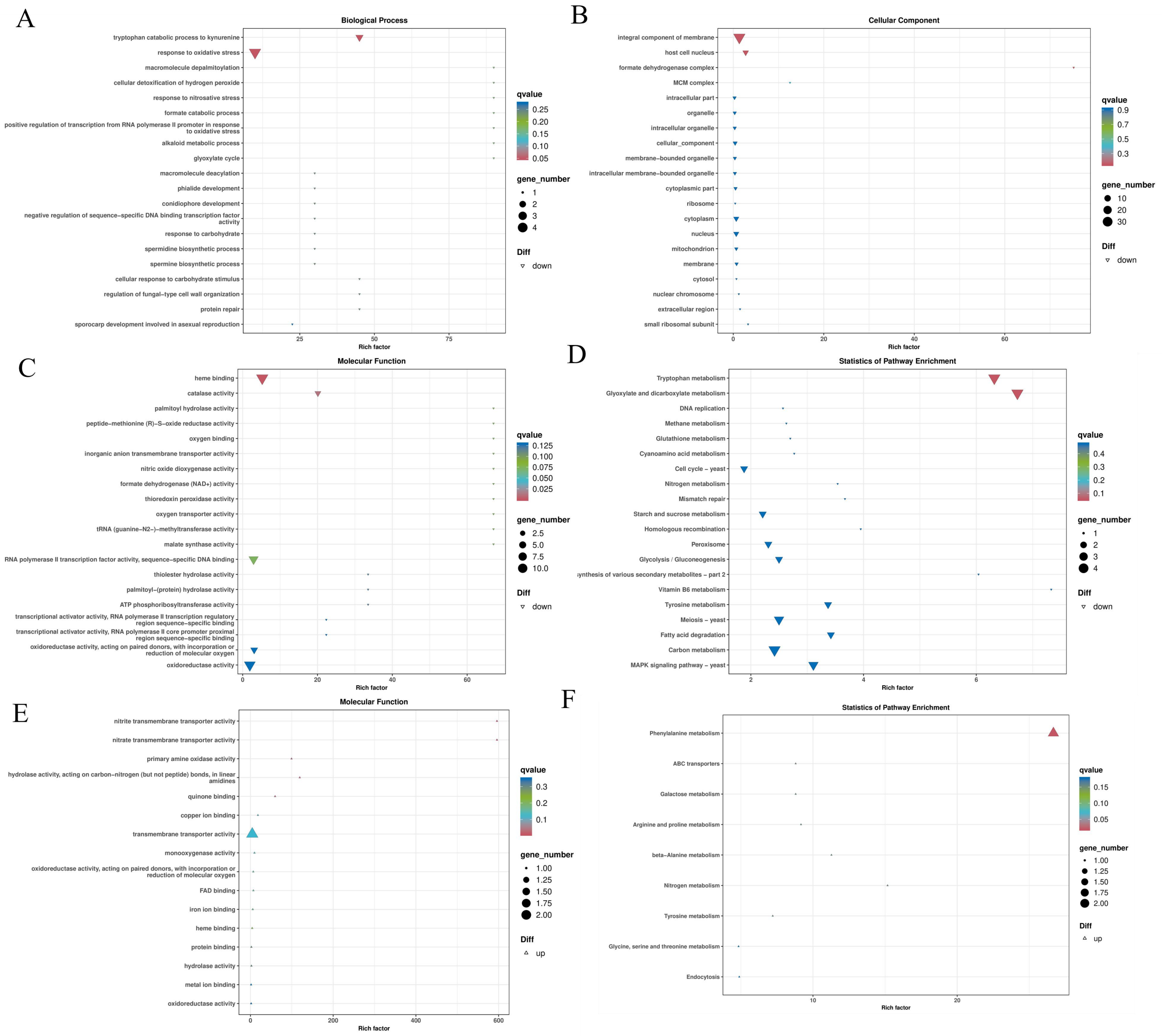

FIG S2 Metabolomic analysis of WT and silenced strains 7-1**. (A-C)** The down-regulated differentially expressed genes GO enrichment analysis in silenced strain 7-1 vs WT. **(D)** The down-regulated differentially expressed genes KEGG enrichment analysis in silenced strain 7-1 vs WT. **(E)** The up-regulated differentially expressed genes KEGG enrichment analysis in silenced strain 7-1 vs WT. **(F)** The up-regulated differentially expressed genes GO enrichment analysis in silenced strain 7-1 vs WT.

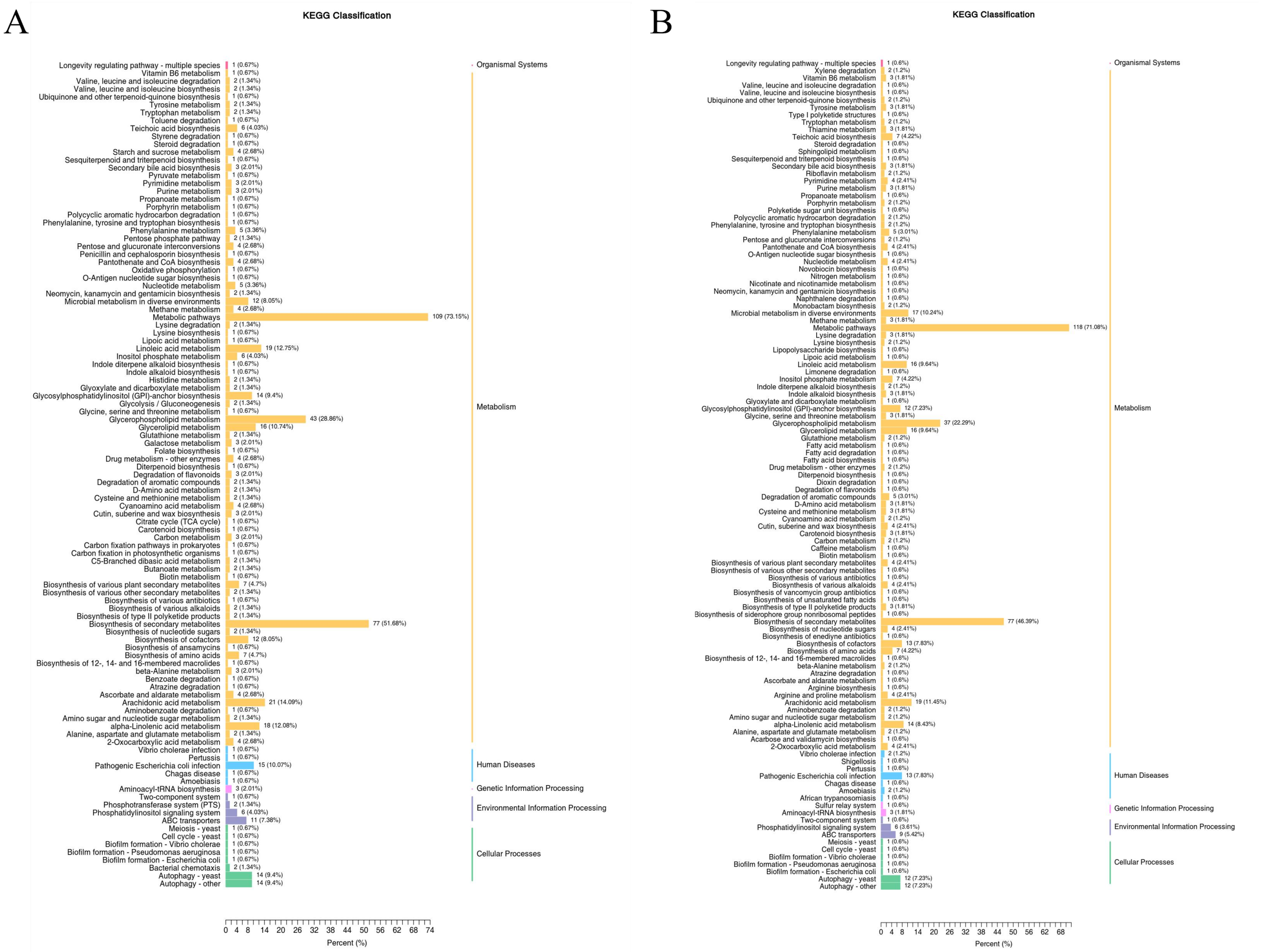

Fig S3 Transcriptomic analysis of WT and silenced strains 3-1 and 7-1. **(A)** KEGG-barplot of differential metabolites between silenced strains WT and 3-1. **(B)** KEGG-barplot of differential metabolites between silenced strains WT and 7-1.

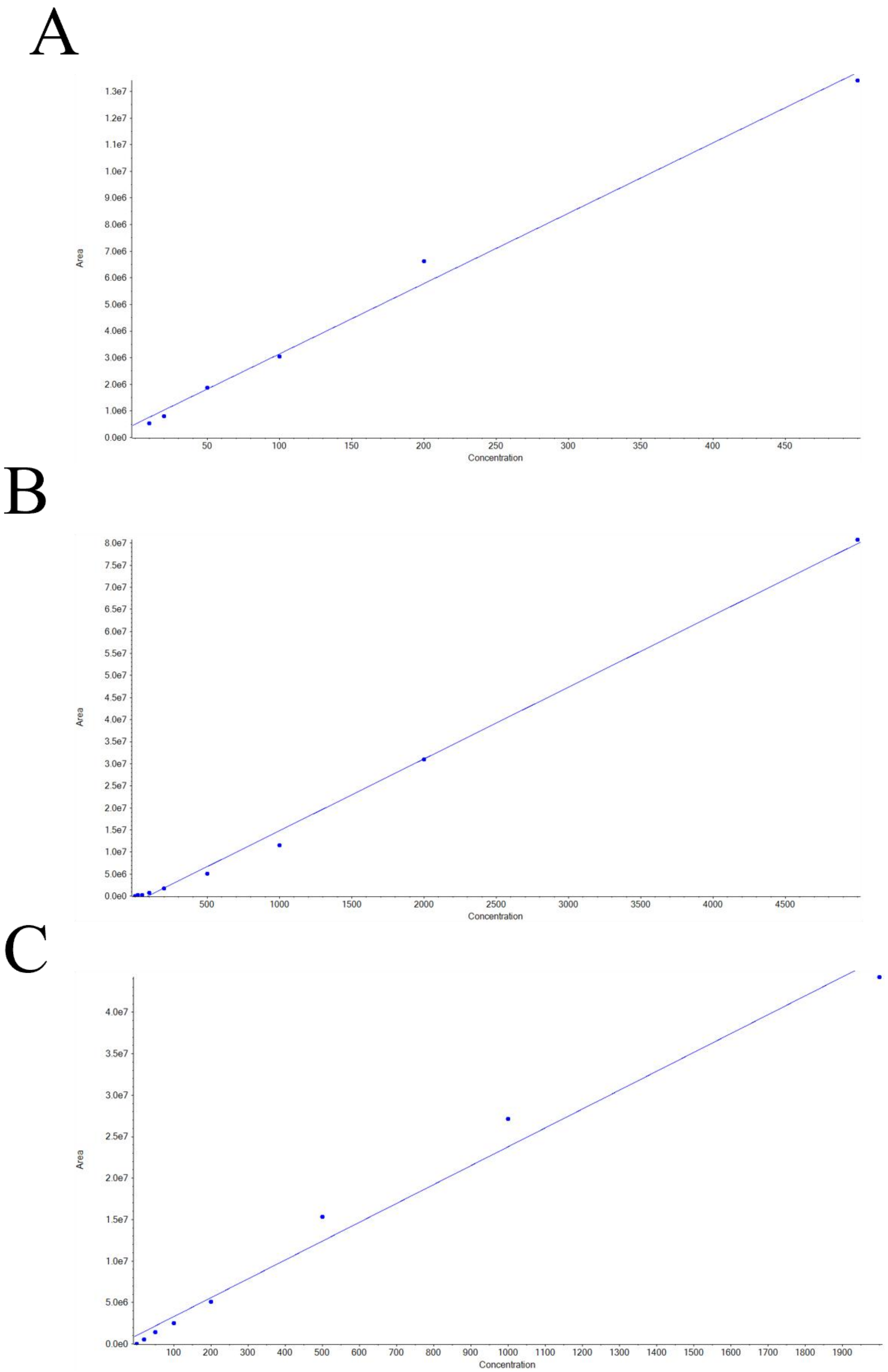

Figure S4 Standard curve of SW, Lys, Pa

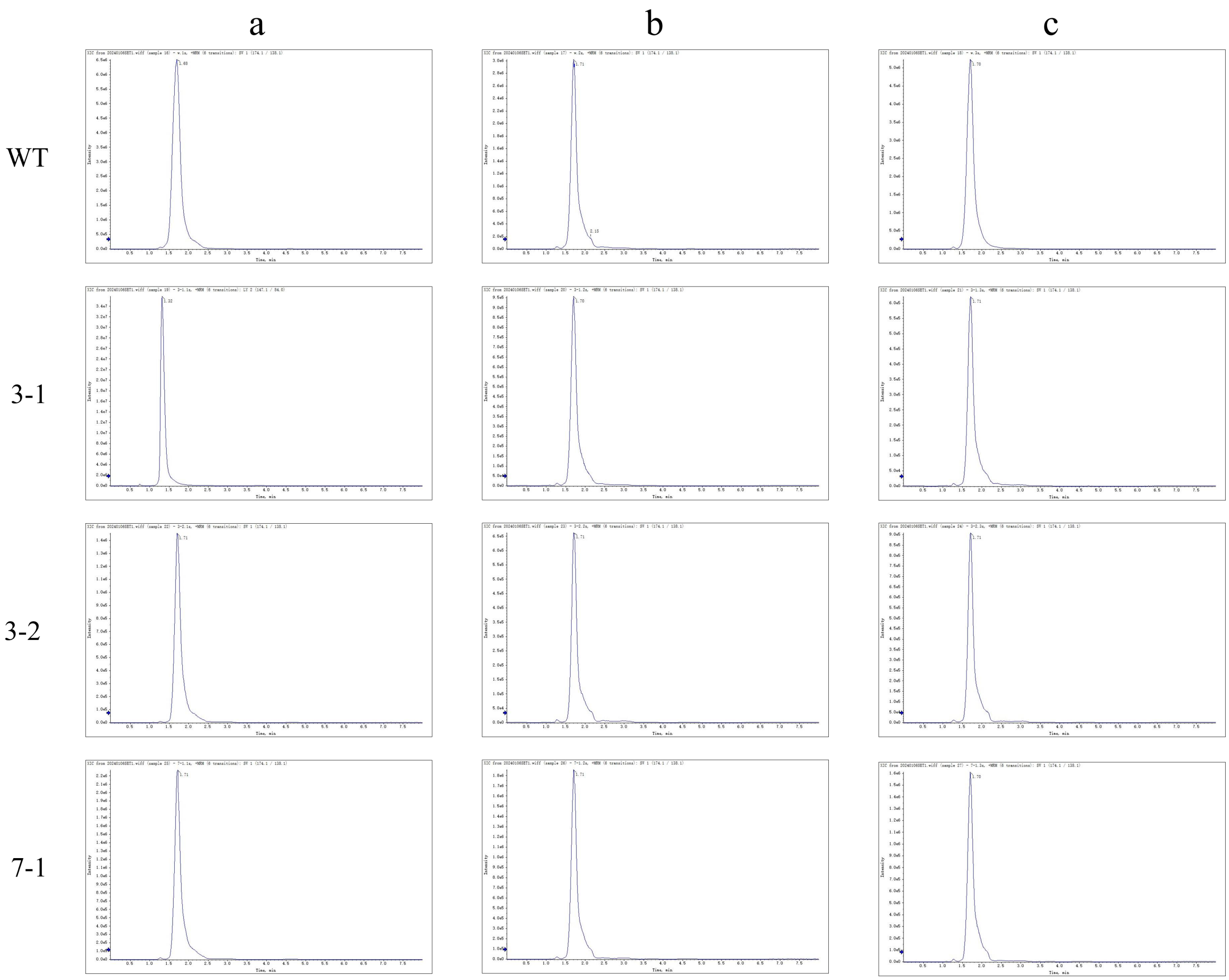

Fig S5 Mass spectrum of SW detection in mycelia of each strain**.** (**A**),(**B**), and (**C**) are three repetitions respectively

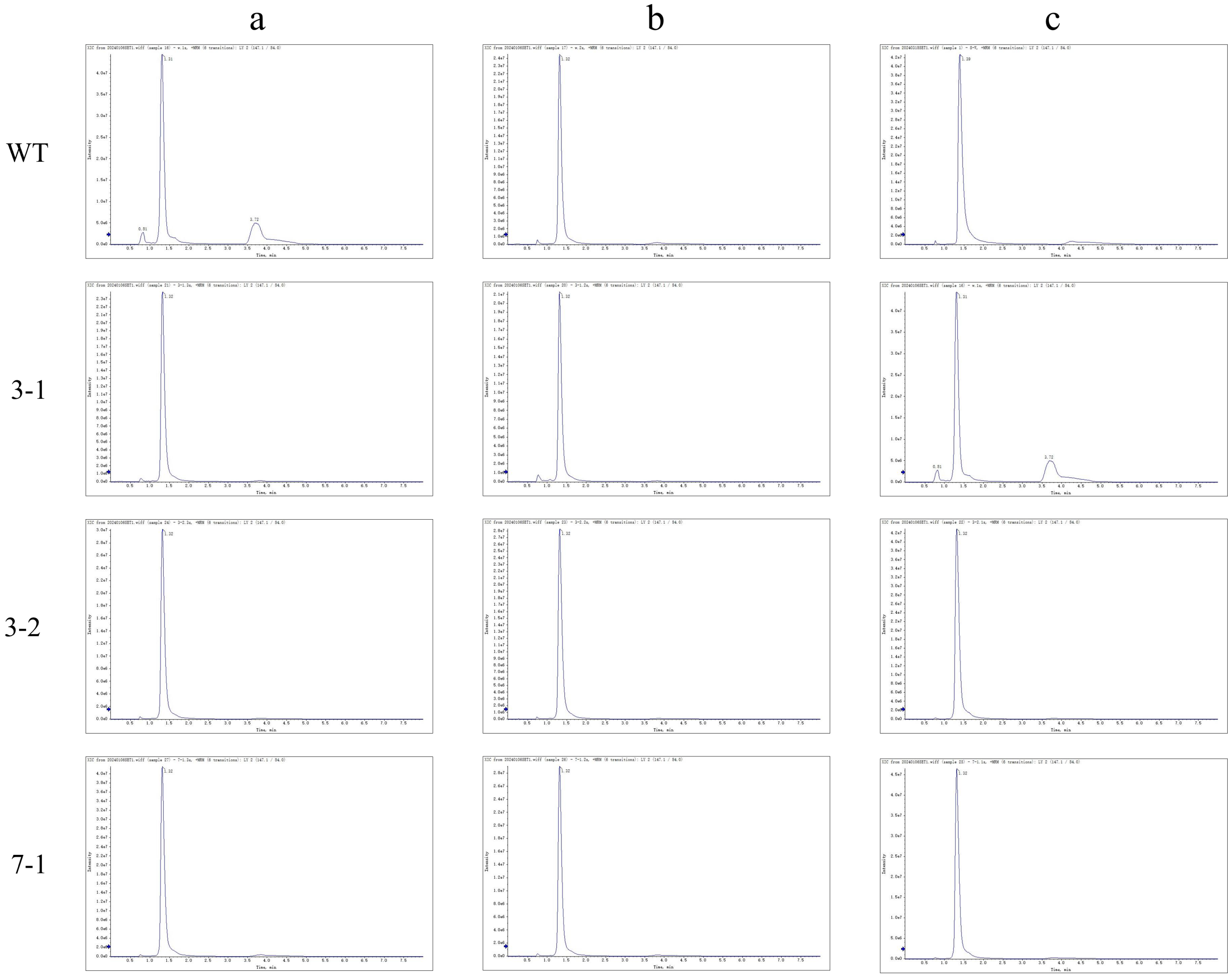

Fig S6 Mass spectrum of Lys detection in mycelia of each strain**.** (**A**),(**B**), and (**C**) are three repetitions respectively

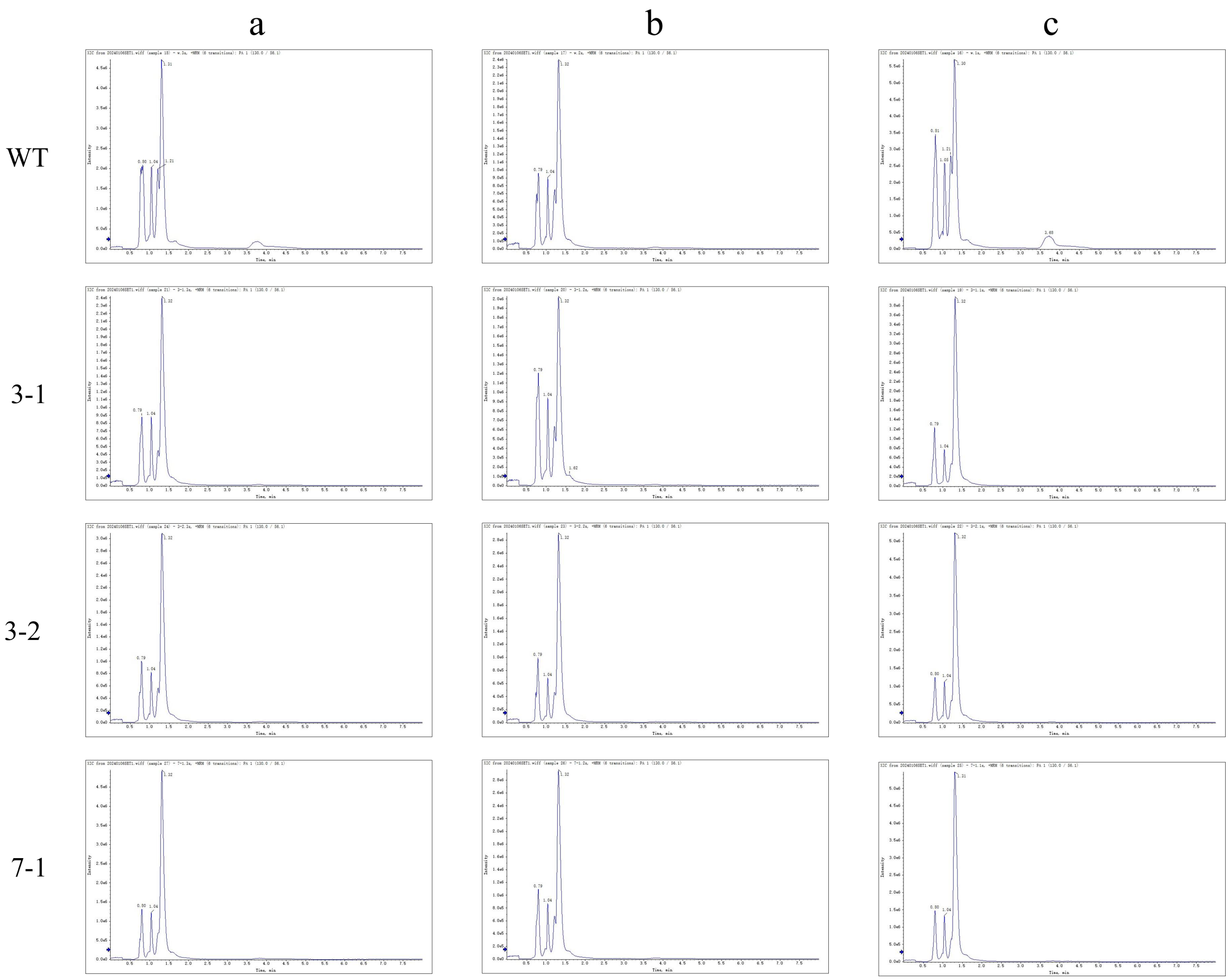

Fig S7 Mass spectrum of Pa detection in mycelia of each strain. (**A**),(**B**), and (**C**) are three repetitions respectively

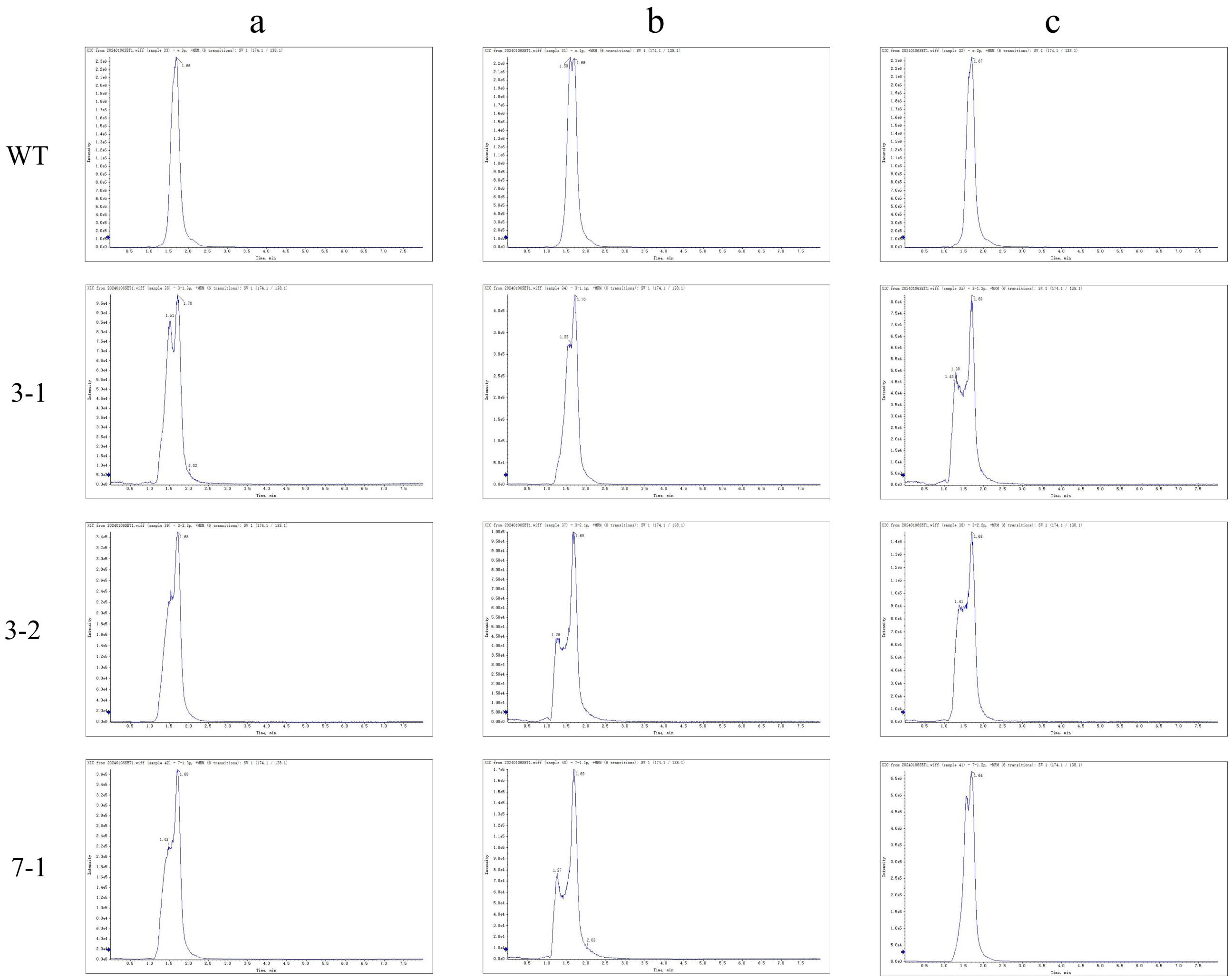

Fig S8 Mass spectrum of SW detection in mycelia of each medium**.** (**A**),(**B**), and (**C**) are three repetitions respectively

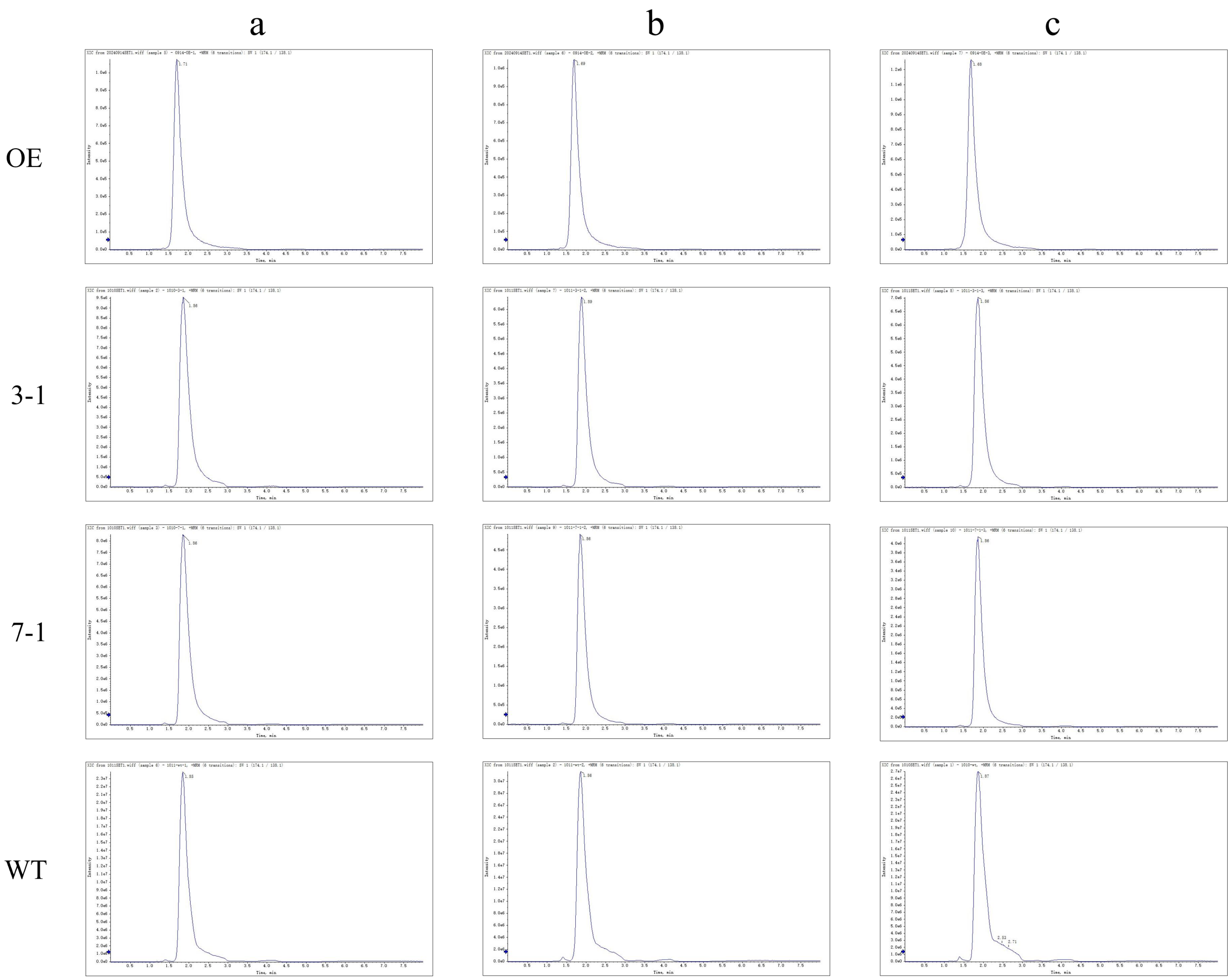

Fig S9 Mass spectrogram of SW content detection in each plant. (**A**),(**B**), and (**C**) are three repetitions respectively**.**

2 Supplementary Tables

Table S1 Statistics of the results of the metabolite difference analysis

Compared Groups	Num. of Total	Num. of Total Sig.	Num. of Sig. Up	Num. of Sig. Down
3-1 vs WT	5668	830	396	434
7-1 vs WT	5668	893	400	493
7-1 vs 3-1	5668	531	189	342

Table S2 List of selected genes primers used for qRT-PCR

Primer	Sequence （5′→3′）
swnH1-F	CTCTATTTCCTCCGCCCAT
swnH1-R	GTGACTTTCCCACAGTTCC
swnH2-F	AGAATGTTGGCGATGGTGT
swnH2-R	GAGGCGTTGTCCTGACTGA
swnN-F	CAACGAACACCCAGAACGG
swnN-R	CAACTTGAGGCAGAAACGG
swnK-F	TCTTTTGCTACCGTCTTTGAAG
swnK-R	GTCGGAAGCGGAACTCTCT
swnR-F	CCTGTTACCCGCCCAATTA
swnR-R	GCCACACACGAACCCATTC
sdh-F	GCCCAACGCTACAACATC
sdh-R	CTCCAAGAAGCCGACCT
p5cr-F	GCGTATTGGTGATGTTGTC
p5cr-R	CTCGCATGGATTGAGTAG
pks-F	TACACTGGCGTCTGGATA
pks-R	CGGGAAGTAGTTTGGGTC
p450-F	ACATTGCCCTCCTTTACC
p450-R	TGCGCCAGTCACGAACA
ITS-F	AGCGAAATGCGATAAGTAATG
ITS-R	GACGGCTGTGCTGGAAAA
HygB-F	CGCAAGGAATCGGTCAATACA
HygB-R	TACACAGCCATCGGTCCAG

TablesS3 Primers for the construction of silence vector pSilent-*swnR*

Primer	Sequence （5′→3′）	Remarks
swnR-Kpn I	ggatccggggcccagGTACCTCAGATTATT	pSilent-*swnR*
swnR-Bgl II	agccagggaacggcagatctCCTTCATGTCCGGAG	
swnR-Xho I	tcgataccgtcgaccTCGAGGTCAGATTAT	
*swnR-Hin*d III	tctccagcaagcttCCTTCATGTCCGGAG	

Table S4 The differential expression table of genes related to swainsonine biosynthesis in transcriptomics

Gene name	ID	WT-1_count	WT-1_FPKM	WT-2_count	WT-2_FPKM	WT-3_count	WT-3_FPKM	3-1-1_count	3-1-1_FPKM	3-1-2_count	3-1-2_FPKM	3-1-3_count	3-1-3_FPKM	7-1-1_count	7-1-1_FPKM	7-1-2_count	7-1-2_FPKM	7-1-3_count	7-1-3_FPKM	WT vs 3-1 DESeq2_log2FC	WT vs 7-1 DESeq2_log2FC
swnR	TRINITY_DN2817_c1_g1	3348	150.09	4996	237.52	6614	327.07	7318	751.08	6368	653.70	7420	761.70	5613	208.48	7553	329.94	8431	454.19	-1.60	-0.474
swnN	TRINITY_DN2817_c0_g1	2071	97.62	2882	144.02	3144	163.35	3244	839.39	3856	1033.41	3541	949.00	3453	39.05	3498	39.57	3615	40.89	-2.837	-1.322
swnK	TRINITY_DN7706_c0_g1	229	1.58	425	3.14	470	3.58	432	1.04	496	1.20	511	1.24	384	291.42	520	394.68	454	344.61	-1.251	-0.1265
SwnH1	TRINITY_DN8537_c0_g1	5199	149.27	8346	254.82	9220	291.96	9227	266.41	9205	282.23	10244	324.48	9672	49.42	12141	62.04	10202	52.12	-1.411	-2.059
SwnH2	TRINITY_DN8537_c0_g1	5199	149.27	8346	254.82	9220	291.96	9227	266.41	9205	282.23	10244	324.48	9672	49.42	12141	62.04	10202	52.12	-1.411	-2.059
P5CR	TRINITY_DN1901_c0_g1	1762	115.44	1703	117.89	1738	125.65	1952	102.16	2356	124.93	1926	102.10	1958	29.76	2198	33.41	2603	39.57	-0.12515	-1.786

Table S5 The differential metabolites in the metabolomics related to swainsonine biosynthesis

Mode	Index	Compounds	WT-1	WT-2	WT-3	WT-4	WT-5	WT-6	3-1-1	3-1-2	3-1-3	3-1-4	3-1-5	3-1-6	7-1-1	7-1-2	7-1-3	7-1-4	7-1-5	7-1-6
T3_negative	MW0169369	L-Lysine	4.15E+05	2.82E+05	3.37E+05	3.22E+05	3.97E+05	3.11E+05	4.44E+05	3.49E+05	2.91E+05	3.17E+05	4.27E+05	3.79E+05	3.02E+05	4.53E+05	3.22E+05	3.46E+05	3.44E+05	4.25E+05
T3_positive	MEDN1024	Piperidine acid	2.27E+03	1.81E+03	6.25E+02	2.77E+03	6.61E+02	2.32E+03	1.36E+03	8.36E+02	1.82E+03	1.21E+03	1.06E+03	1.19E+03	2.73E+03	3.16E+03	1.94E+03	1.19E+03	5.50E+02	7.75E+02
T3_negative	FDATN01488	L-Glutamic acid	9.38E+05	1.10E+06	7.33E+05	1.18E+06	7.55E+05	8.02E+05	1.32E+06	1.27E+06	1.41E+06	8.98E+05	1.09E+06	6.36E+05	9.63E+05	6.30E+05	7.61E+05	8.20E+05	9.10E+05	5.16E+05
T3_positive	MEDP0058	L-Saccharopine	8.20E+05	7.00E+05	5.68E+05	7.80E+05	6.90E+05	4.49E+05	4.01E+05	4.98E+05	6.66E+05	4.01E+05	5.12E+05	6.04E+05	3.12E+05	5.92E+05	2.05E+05	6.13E+05	4.43E+05	1.86E+05
T3_negative	MW0169367	L-Proline	1.67E+04	1.24E+04	1.14E+04	1.01E+04	1.39E+04	1.41E+04	1.28E+04	1.17E+04	1.25E+04	1.18E+04	1.06E+04	1.58E+04	1.25E+04	1.04E+04	1.18E+04	1.36E+04	1.50E+04	1.16E+04
